# Pharmacokinetics and Metabolism of Liposome-Encapsulated 2,4,6-Trihydroxygeranylacetophenone in Rats Using High-Resolution Orbitrap Liquid Chromatography Mass Spectrometry

**DOI:** 10.3390/molecules25133069

**Published:** 2020-07-06

**Authors:** Yamen Alkhateeb, Qais Bashir Jarrar, Faridah Abas, Yaya Rukayadi, Chau Ling Tham, Yuen Kah Hay, Khozirah Shaari

**Affiliations:** 1 Laboratory of Natural Product, Institute of Bioscience, Universiti Putra Malaysia, Serdang 43400, Selangor, Malaysia; yamen.alkhatib84@gmail.com; 2 Department of Applied Pharmaceutical Sciences, Faculty of Pharmacy, Al-Isra University, Amman 11622, Jordan; jarrarq@yahoo.com; 3 Department of Food Science, Faculty of Food Science and Technology, Universiti Putra Malaysia, Serdang 43400, Selangor, Malaysia; faridah_abas@upm.edu.my (F.A.); yaya_rukayadi@upm.edu.my (Y.R.); 4 Department of Biomedical Science, Faculty of Medicine & Health Sciences, Universiti Putra Malaysia, Serdang 43400 UPM, Selangor, Malaysia; chauling@upm.edu.my; 5 School of Pharmaceutical Sciences, Universiti Sains Malaysia, Glugor 11800, Penang, Malaysia; khyuen@usm.my

**Keywords:** pharmacokinetics, metabolism, trihydroxygeranylacetophenone, liposomes, LC-HRMS method validation

## Abstract

2,4,6-trihydroxy-3-geranylacetophenone (tHGA) is a bioactive compound that shows excellent anti-inflammatory properties. However, its pharmacokinetics and metabolism have yet to be evaluated. In this study, a sensitive LC-HRMS method was developed and validated to quantify tHGA in rat plasma. The method showed good linearity (0.5–80 ng/mL). The accuracy and precision were within 10%. Pharmacokinetic investigations were performed on three groups of six rats. The first two groups were given oral administrations of unformulated and liposome-encapsulated tHGA, respectively, while the third group received intraperitoneal administration of liposome-encapsulated tHGA. The maximum concentration (C_max_), the time required to reach C_max_ (t_max_), elimination half-life (t_1/2_) and area under curve (AUC_0–24_) values for intraperitoneal administration were 54.6 ng/mL, 1.5 h, 6.7 h, and 193.9 ng/mL·h, respectively. For the oral administration of unformulated and formulated tHGA, C_max_ values were 5.4 and 14.5 ng/mL, t_max_ values were 0.25 h for both, t_1/2_ values were 6.9 and 6.6 h, and AUC_0–24_ values were 17.6 and 40.7 ng/mL·h, respectively. The liposomal formulation improved the relative oral bioavailability of tHGA from 9.1% to 21.0% which was a 2.3-fold increment. Further, a total of 12 metabolites were detected and structurally characterized. The metabolites were mainly products of oxidation and glucuronide conjugation.

## 1. Introduction

The chemical diversity of natural products is more complex than any chemical library made by humans, representing a massive reservoir of valuable molecules. As history has shown, it has yielded a huge number of drug leads, many of which have been successfully developed into clinical drugs. Despite a period of declining use of natural products in drug discovery screening, triggered by perceived disadvantages of natural products (access and supply, intellectual property rights, complex chemistry and inherent slowness of working with natural products), pharmaceutical companies are turning their interests back to natural product compounds, which clearly shows that they still command a crucial role in healthcare. There are better opportunities to explore their biological activities and many of the associated technical drawbacks in natural products research have been lessened. For example, developments in chemical synthesis technologies are now able to overcome the challenges in the isolation and purification of active compounds from complex extracts and made it available in sufficient amounts. The aromatic compound 2,4,6-trihydroxy-3-geranylacetophenone (tHGA) was originally a natural and bioactive compound isolated from the leaves of *Melicope ptelefolia* [1]. Our research group has demonstrated its anti-inflammatory properties, including the airway inflammation, with a potency comparable to clinically used drugs (Zileuton and Ketotifen), in several in vitro and in vivo studies [2,3,4,5,6,7,8,9]. The dual COX-2/5-LOX inhibition activity of tHGA makes it a promising anti-inflammatory and anti-allergic drug with fewer adverse effects [10].

Drug metabolism and pharmacokinetics (DMPK) play a sensitive and essential role in the process of drug discovery and development. Pharmacokinetics is proposed to study the fate of drugs in the body over a period of time, including the bodily absorption, distribution, metabolism, and excretion of drugs in vivo [11]. Information on the pharmacokinetics, as well as the metabolic products of a drug, is crucial in the assessment and understanding of the drug candidate’s efficacy and safety. In addition, it is important to reduce the clinical failures related to poor pharmacokinetic properties or toxic metabolites. The quality of DMPK studies is highly correlated with the quality of the bioanalysis data produced. High-resolution LC-MS technologies have been the preferred tool to identify and quantify the drug and its metabolites in the complex matrix due to its superior sensitivity and specificity [12].

In this study, a bioanalytical LC-MS method was developed and validated to quantify tHGA in rat plasma. The method was successfully applied to evaluate the pharmacokinetic properties of tHGA after oral (PO) and intraperitoneal (IP) administration to rats. Furthermore, a non-target screening method was used to identify the metabolites of tHGA in rat plasma, urine, and feces using a high-resolution orbitrap UHPLC-MS system. To the best of our knowledge, there has not been any bioanalytical method developed to quantify tHGA in plasma and nothing is known about the pharmacokinetics and the metabolism of tHGA. This information is a must-have for a better insight into its clinical potential.

## 2. Results

### 2.1. Method Validation

**Figure 1:**
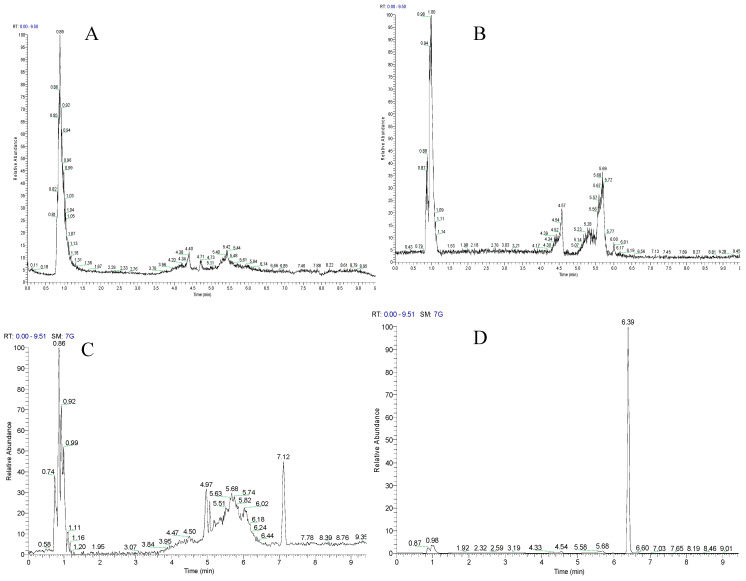
Base peak chromatograms of plasma, after protein precipitation and extraction, analyzed in negative ion mode; (**A**) blank plasma and (**C**) treated plasma, for the 302.5–303.5 mass range revealing no interference with tHGA at RT 7.1 min, (**B**) blank plasma and (**D**) treated plasma sample showing for the 204.5–205.5 mass range revealing no interference with the IS at RT at 6.3 min. Treated plasma was obtained from rat blood spiked with 0.5 ng/mL of tHGA.

As shown in Figure 1, comparison between the chromatogram of blank plasma samples, and the chromatogram of plasma samples spiked with 0.5 ng/mL of tHGA.

(lower limit of quantification, LLOQ), after protein precipitation and extraction, illustrated that there was no significant interference from endogenous compounds of rat plasma observed at the retention times of tHGA (7.1 min) and the internal standard IS (6.3 min) which was used to minimize the matrix and ionization effects [13].

The calibration curves exhibited good linearity based on three individual calibration curves constructed on three different days over a concentration range of 0.5 to 80 ng/mL of tHGA. The mean linear regression equation obtained for the calibration curves were y = 0.0098x + 0.0007 (day one), y = 0.0137x + 0.0017 (day two) and y = 0.0138x + 0.002 (day three) with coefficient of determinations (R^2^) of 0.9995, 0.9967 and 0.9971, respectively. The results showed a good relationship between tHGA concentration and the response of the LC-MS system which proved the ability of this method to accurately measure an unknown concentration of tHGA in plasma samples. The LLOQ value, which is the lowest concentration that can be quantified with accuracy and precision of less than 20%, was 0.5 ng/mL. This low LLOQ value revealed the excellent sensitivity of the method and its suitability for the pharmacokinetic study.

As shown in Table 1, the within-run and between-run accuracies (RE) were less than 9.4% and 7.2%, respectively. The within-run and between-run precisions (RSD) were less than 6.8% and 8.7%, respectively. The results were within the tolerable values (±20% for LLOQ and ±15% for low quality control (LQC), medium quality control (MQC), and high quality control (HQC) samples) demonstrating that the developed bioanalytical method is accurate and precise for quantifying tHGA in rat plasma.

The extraction recovery and matrix effects (ME) were estimated in six replicates of three QC levels (LQC, MQC, and HQC). As shown in Table 2, the matrix effect values for tHGA were within the acceptable range of 91.4–107.8%. The low matrix effect values proved that the effect of plasma on accuracy and precision of the analysis of tHGA was tolerable and thus, the method is reliable for the determination of tHGA concentrations in plasma. The recoveries of tHGA and the IS ranged between 96.3–100.3% and 95.6–99.6%, respectively. These results indicated that ACN is an efficient solvent to extract tHGA and IS from rat plasma, giving consistent results.

Dilution integrity was performed in five replicates after ten-fold dilution of a high concentration (500 ng/mL) sample of tHGA in rat plasma. The mean accuracy (RE%) and precision (RSD%) were −5.3% and 9.0%, respectively. Carry-over was evaluated by injecting blank plasma samples in duplicates after ULOQ. Following this, the peak areas of tHGA in the blank were compared with the peak area of LLOQ. The mean carry-over percentage was 6.3% ± 2.8. The results demonstrated that the impact of the dilution on the accuracy and precision of the analysis for samples with a concentration higher than ULOQ was within the range and the measured concentration was not changed due to residual tHGA from a preceding sample.

The short- and long-term stability studies for tHGA were conducted for LQC and HQC samples (n = 6), and the data are summarized in Table 2. The QC plasma samples were stable for at least 24 h in the autosampler set at 4 °C post sample preparation with accuracy from −4.6% to 5.3%. The QC plasma samples for long-term storage of 1 month at −80 °C had an accuracy of 5.3–5.9%.

### 2.2. Pharmacokinetic Study

**Figure 2:**
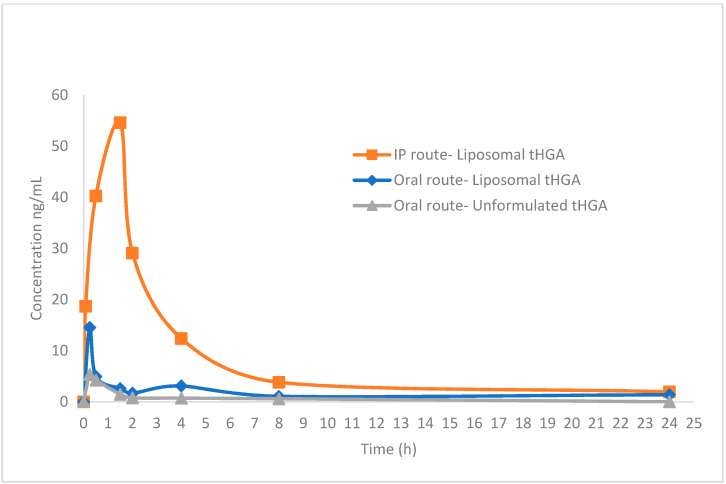
Plasma tHGA concentrations versus time profile for oral administration of liposomal formulation, oral administration of free tHGA, and intraperitoneal administration of tHGA, at a dose of 20 mg/kg. Data are represented as mean value (n = 6).

Table 3 presents the basic pharmacokinetic parameters estimated from the non-compartmental module using ThothPro software, following PO administrations of 20 mg/kg free tHGA, and PO and IP administration of 20 mg/kg liposome-encapsulated tHGA, in six male S.D rats per group. For PO administration, C_max_ values for the free tHGA and liposome-encapsulated tHGA were calculated to be 5.4 ng/mL and 14.5 ng/mL, respectively. For the IP administration of tHGA-loaded liposomes, the C_max_ value was 54.6 ng/mL. The t_max_ values for free tHGA and liposome-encapsulated tHGA after PO administration were the same, i.e., 0.25 h, while for IP administration the value was 1.5 h. The AUC_0–24_ for free tHGA and liposome-encapsulated tHGA after PO administration and IP administration were 17.6, 40.7, and 193.9 ng·h/mL, respectively. Clearer graphical pharmacokinetic profiles are as presented in Figure 2.

The software SPSS was used to compare the values of AUC_0–24_ for the free and encapsulated tHGA administrated orally. The U-test revealed that there is a significant difference between the AUC_0–24_ values for the free and encapsulated tHGA (*p* < 0.05). On the other hand, no significant differences were observed for C_max_, t_max,_ and t_1/2_ values between the free and the encapsulated tHGA. The AUC_0–24_ value for encapsulated tHGA increased by 2.3-fold compared to the free tHGA. The group administrated intraperitoneally with liposome-encapsulated tHGA showed significant differences in t_max_, C_max_ and AUC_0–24_ values. The C_max_ value for the IP group was 3.8-fold and 10.2-fold greater than the C_max_ value for the encapsulated and free tHGA, respectively. The differences in AUC_0–24_ values were exemplified by the relative oral bioavailability values. The relative oral bioavailabilities of the free and encapsulated tHGA were 9.1% and 21.0%, respectively. The relative oral bioavailability of the liposomal formulation of tHGA increased by 2.3-fold compared to free tHGA. Despite the low oral bioavailability, the therapeutic activity of tHGA in pre-clinical trials has been demonstrated, which indicated the high efficacy of the compound.

Several studies have demonstrated that the liposomal drug delivery system can enhance the therapeutic efficacy and reduce drug toxicities, which were attributed to their ability to enhance the bioavailability of poorly water-soluble drugs [14,15,16,17,18]. Liposomes can improve the bioavailability of drugs generally by enhancing their absorption, protecting the drugs from rapid metabolism, and prolonging biological half-life [19]. The small particle size and thus, the larger surface area of the prepared liposomal formulation of tHGA, increased the solubility and the absorption of tHGA which led to the improved AUC_0-24_ value and bioavailability after PO administration, in comparison to the free tHGA. However, despite these improvements, the C_max_ value of tHGA was still low. The low concentration of tHGA in plasma samples might be due to degradation of the formulation in the gastrointestinal tract which limited its ability to protect tHGA from rapid elimination. On the other hand, the C_max_ value after IP administration of tHGA-loaded liposomes was significantly higher than the C_max_ value after PO administration. These values indicated that the IP route is the preferable route to maximize tHGA concentrations in the blood and improve the pharmacokinetic profile of the liposomal formulation of tHGA.

### 2.3. Metabolite Identification

Identification of the parent drug was based on the peak eluted at RT 25.7 min (Figure S1) which gave [M − H]^–^ and [M + H]^+^ ions *m*/*z* 303.1602 (Figure S2A) and 305.1762 (Figure S3A), respectively. The identification was further supported by its mass fragmentation in the negative and positive ion modes shown in Figures S2B and S3B, respectively.

Metabolite M1 was eluted at 20.5 min (Figure S4). It was identified as a glucuronide conjugation product of tHGA based on its [M − H]^−^ ion at *m*/*z* 479.1918 (Figure S5A) and [M + H]^+^ ion at *m*/*z* 481.2091 (Figure S6A). The identity of the metabolite was confirmed by matching the MS/MS fragmentation spectra of M1 and the parent compound. The comparison showed five mutual fragment ions in negative ion mode (Figure S5B) and five mutual fragment ions in positive ion mode (Figure S6B). The glucuronide conjugation reaction is catalyzed by UDP-glucuronyltransferases (UGT) enzymes [20]. Hence, UGT catalyzed the biotransformation of tHGA to M1. The site of metabolism is expected to be on the hydroxyl group on the C-5 position of the aromatic ring.

Metabolite M2 and its positional isomer M3 were eluted at 18.8 and 21.2 min, respectively (Figure S7). The two positional isomers were identified as hydroxylation (oxidation) products of tHGA based on their common molecular ions at *m*/*z* 319.1543 in the negative ion mode (Figures S8A and S9A). The identity of the metabolites was confirmed by comparing the MS/MS fragmentation spectra of M2 structural formulas and M3 with tHGA in the negative ion mode as shown in Figures S8B and S9B, respectively. The comparison between M2 and tHGA showed almost identical fragmentation patterns with 13 mutual fragments, while M3 and tHGA showed five mutual fragments. Hydroxylation of aliphatic groups is very common in drug metabolism and the reaction is catalyzed by cytochromes P450 (CYP) enzymes (mostly CYP3A4) [20]. Thus, CYP catalyzed the biotransformation of tHGA to M2 and M3. Hydroxylation can occur at primary, secondary, and tertiary carbon atoms [21]. The site of metabolism is proposed to be at the secondary carbon atoms, C-18 and C-13 to form M2 and M3, respectively.

Metabolite M4 was eluted at 19.1 min (Figure S10). It was identified as the dihydroxylation product of tHGA or a hydroxylated metabolite of M2 based on its molecular ions at *m*/*z* 335.1493 and 337.1659 in the negative (Figure S11A) and positive (Figure S12A) ion modes, respectively. The identity of the metabolite was confirmed by comparing the MS/MS fragmentation spectra of M4 (Figures S11B and S12B) with the fragmentation spectrum of tHGA and M2. The comparison with tHGA showed five and six mutual fragment ions in negative and positive ion modes, respectively. The comparison with M2 showed six mutual fragment ions in negative ion mode. CYP catalyzed the biotransformation of tHGA to M4 (or M2 to M4) and the site of metabolism is expected to be at the secondary carbon atoms C-13 and C-18.

Metabolite M5 was eluted at 19.8 min (Figure S13). M5 was identified as a hydrolysis product of tHGA based on its molecular ion at *m*/*z* value at 321.1700 in negative ion mode (Figure S14A). The identity of the metabolite was confirmed by comparing the MS/MS fragmentation spectra of M5 (Figure S14B) and tHGA. The comparison showed nine mutual fragment ions. The hydroxylation at tertiary carbon atoms is the preferable oxidation site to metabolize aliphatic carbon atoms (Markey, 2012), it could be accomplished with desaturation as in the case of tHGA. CYP catalyzed the biotransformation of tHGA to M5 and the site of metabolism is expected to be the tertiary carbon (C-19) in the geranyl substituent.

Metabolite M6 was eluted at 19.0 min (Figure S15). It was identified as a desaturation product of M4 based on its molecular ion at *m*/*z* values 333.1337 in negative ion mode (Figure S16A). The identity of the metabolite was confirmed by comparing the MS/MS fragmentation spectra of M6 (Figure S16B) with the fragmentation spectrum of tHGA and M4. The comparison with tHGA in negative ion mode showed eight mutual fragment ions. The comparison with M4 showed an almost identical fragmentation pattern when subtracting two hydrogen atoms. CYP catalyzed the biotransformation of M4 to M6 and the site of desaturation is proposed to be on the carbons C-16 and C-17 of the geranyl substituent.

Metabolite M7 was eluted at 25.4 min (Figure S17). M7 was identified as a desaturated product of tHGA based on its molecular ion at *m*/*z* 301.1438 in negative ion mode (Figure S18A). The comparison between MS/MS fragmentation spectra of M7 (Figure S18B) and tHGA showed six mutual fragment ions. The desaturation of aliphatic groups to form olefins by CYP is a common transformation in drug metabolism [22]. CYP catalyzed the biotransformation of tHGA to M7 and the site of desaturation is proposed to be between the two methylene groups at C-16 and C-17 of the geranyl substituent.

Metabolite M8 was eluted at 17.04 min (Figure S19). It was identified as glucuronidation product of M2 (hydroxylation and glucuronidation) based on the molecular ion at *m*/*z* 495.1865 in negative ion mode (Figure S20A). M8 was also detected in the positive ion mode but without MS/MS fragmentations due to the very low intensity. The comparison between MS/MS fragmentation spectra of M8 (Figure S20B) and M2 showed five mutual fragment ions with major fragment ion at *m*/*z* 319.1548 which is the *m*/*z* value of M2. The more probable biotransformation pathway, in this case, could be glucuronidation of M2 because generally, glucuronidation takes place last in most cases of biotransformation. However, there are some cases were a number of xenobiotic compounds have been reported to undergo oxidation after glucuronidation [23,24]. Thus, M8 could still result via hydroxylation of M1.

Metabolite M9 was eluted at 15.8 min (Figure S21). It was identified as the sulfation product of M4 based on its molecular ion at *m*/*z* values at 415.1066 in negative ion mode (Figure S22A). The comparison between MS/MS fragmentation spectra of M9 (Figure S22B) with tHGA and M4 in negative ion mode showed four and ten mutual fragment ions, respectively. Sulfation is a phase-II reaction catalyzed by sulfotransferases enzymes that can metabolize phenols to sulfate esters [21]. Consequently, SULTs catalyzed the biotransformation of M4 to M9 and the site of sulfation is proposed to be on the hydroxyl group on the C-5 position of the aromatic ring.

Metabolite M10, eluted at 15.5 min (Figure S23), was identified as the hydroxylated product of M5 based on its molecular ion at an *m*/*z* value at 337.1650 in negative ion mode (Figure S24A). The comparison between the MS/MS fragmentation spectra of M10 (Figure S24B) with tHGA and M5 showed nine mutual fragments ion for both of them. CYP catalyzed the hydroxylation of M5 to M10 and the site of metabolism is proposed to be on the secondary carbon C-18 on the geranyl substituent.

Metabolite M11, eluted at 17.8 min (Figure S25), was identified as a glucuronidation product of M5 based on the molecular ion at *m*/*z* 497.2022 in negative ion mode (Figure S26A). The comparison between the MS/MS fragmentation spectra of M11 (Figure S26B) and M5 showed six mutual fragment ions with a very high intensity of at *m*/*z* 321.1703 which match the *m*/*z* value of M5. The site of metabolism is proposed to be on the hydroxyl group on C-5 of the aromatic ring.

Metabolite M12, eluted at 15.8 min (Figure S27), was identified as a glucuronidation product of M4 based on its molecular ion at *m*/*z* 511.1813 in negative ion mode (Figure S28A). The comparison between the MS/MS fragmentation spectra of M12 (Figure S28B) and M4 showed four mutual fragment ions with a major fragment at *m*/*z* 335.1496, which matches the *m*/*z* value of M4. The site of metabolism is proposed to be on the hydroxyl group on C-5 of the aromatic ring.

**Figure 3:**
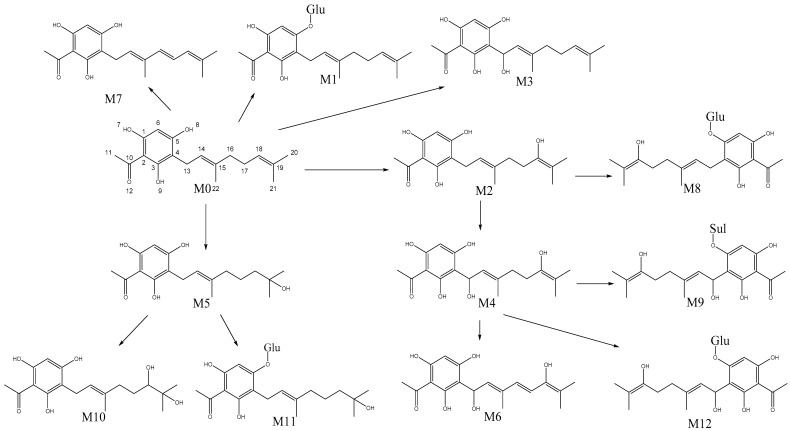
Proposed metabolism pathways of tHGA in rats.

The mass spectral characteristics of tHGA (M_0_) and its metabolites in the blood, urine, and feces of the experimental animals are summarized in Table 4, and the proposed metabolism pathways of the metabolites are presented in Figure 3.

## 3. Materials and Methods 

### 3.1. Chemicals and Reagents

tHGA was synthesized, purified, and fully characterized by suitable spectroscopic techniques in our laboratory (unpublished manuscript). The purity of tHGA was 98.4% as determined by qNMR. Acetonitrile, water and formic acid (LC-MS grade) were purchased from Fisher Scientific (Pittsburgh, PA, USA). Ibuprofen was purchased from Sigma-Aldrich (St. Louis, MO, USA). Ultrapure water for HPLC was taken from a Sartorius Arium 611DI ultra-pure water purification system (Sartorius Stedim Biotech, Goettingen, Germany). Prolipo^TM^ Duo was purchased from Lucas Meyer (Le Blanc Mesnil, France).

### 3.2. Animals, Dosing and Sampling

Thirty healthy male Sprague-Dawley (S.D.) rats, weighing between 180 and 225 g on the day of dosing, were kept in polypropylene cage base with stainless steel wire top clips for three days before the experiment. The living environment of the animals was controlled as follows: 12/12-h dark/light cycle, temperature maintained between 22 to 25 °C, daily feeding with commercial pellet food, and tap water. Rats were fasted 12 h before dosing and labeled with a designated number at the base of their tails. The rats were then randomly divided into six groups as follows:

- Group 1 (n = 6) were dosed orally with 20 mg/kg of free tHGA.
- Group 2 (n = 6) were dosed orally with 20 mg/kg of liposomes-encapsulated tHGA.
- Group 3 (n = 6) were dosed intraperitoneally with 20 mg/kg of liposomes-encapsulated tHGA
- Group 4 (n = 4) were dosed orally with 20 mg/kg of liposomes-encapsulated tHGA.
- Group 5 (n = 4, control) were dosed orally with blank liposomes formulation (for urine and feces collection).
- Group 6 (n = 4, control) were dosed orally with blank liposomes formulation (for blood collection).

Groups 1 to 3 were used for the single-dose pharmacokinetic study. An aliquot of 200 µL of blood was drawn from each rat via the tail vein into polypropylene tubes containing a disodium K_2_EDTA solution (10%) as an anticoagulant at the pre-specified time points; at 0.25, 0.5, 1.5, 2, 4, 8 and 24 h for groups 1 and 2 after PO dose, and at 0.08, 0.5, 1.5, 2, 4, 8 and 24 h for group 3 after IP dose. Blood samples were centrifuged at 14,000 rpm at 20 °C for 10 min to separate the plasma which was then stored under −80 °C until further analysis. Animals were allowed to feed two hours after the first sample time point.

Groups 4 to 6 were used for single-dose drug metabolism study. Each rat was kept in individual metabolic cages. Urine and faeces were collected from each rat in the tHGA-treated group 4 and control group 5 at four different times over a period of 24 h. The samples were stored at −80 °C until further analysis. For the determination of plasma metabolites, blood was sampled from each rat in the control group 6, according to the same procedure as in the pharmacokinetic study. Blood samples collected from the tHGA-treated group 2 in the pharmacokinetics study were also used for the metabolism study.

All experiments and animal handling were performed in accordance with the principles and guidelines of animal care and use committee (IACUC) with approval obtained from IACUC (UPM/IACUC/AUP-R079/2018).

### 3.3. Preparation of Solutions 

**Figure 4:**
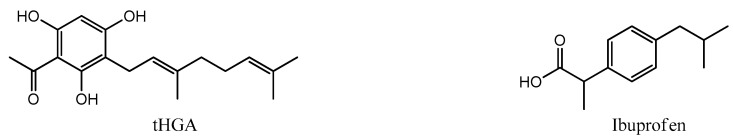
The chemical structures of 2,4,6-trihydroxy-3-geranylacetophenone (tHGA) and the internal standard, ibuprofen.

A freshly prepared stock solution of tHGA was prepared in acetonitrile and appropriately diluted to obtain standard solutions with concentrations ranging from 0.5 to 80 ng/mL. A 5 µL aliquot of each concentration was then added to 45 µL of blank rat plasma and 200 µL acetonitrile containing 1 µg/mL of ibuprofen as an internal standard (Figure 4). The mixture was vortexed for 30 s and centrifuged for 10 min at 14,000 rpm. The supernatant was transferred into LC-MS vials and 10 µL was injected into the LC-MS system for spectral acquisition. Three different concentrations of QC samples representing the entire range of the calibration curve were prepared using the same sample preparation and extraction method, 1.5 ng/mL for low quality control (LQC), 35.0 ng/mL for medium quality control (MQC), and 70.0 ng/mL for high quality control (HQC).

Due to poor water solubility of tHGA, a liposomal formulation of tHGA was prepared from Prolipo Duo according to the manufacturer’s (Lucas Meyer, Le Blanc Mesnilcity, France) instructions with some modification (unpublished manuscript).

### 3.4. Sample Preparation

For the pharmacokinetic study, the same procedure used in the preparation of the standard solutions was used to extract tHGA from plasma samples.

For the drug metabolism study, for each rat, plasma samples collected over a period of 24 h were combined and tHGA was similarly extracted from the samples. For the urine samples, for each rat, samples collected over the 24 h period were combined and centrifuged for 15 min at 14,000 rpm. The supernatant was then vacuum-evaporated at 40 °C to a volume of 5 mL, after which, 30 mL methanol was added, the mixture sonicated for 25 min, followed by centrifuging at 10,000 rpm for 15 min. The supernatant was again vacuum-concentrated at 40 °C, to a volume of 5 mL, filtered through 0.45 μm membrane filter into an LC-MS vial and then injected (10 µL) to UHPLC-MS/MS system for spectral acquisition.

The urine sample collected over 24 h was homogenized with distilled water in the ratio of 1 g to 3 mL and sonicated for 10 min. From this mixture, 3 mL was taken and mixed with 9 mL methanol. After that, the mixture was sonicated for 30 min and then centrifuged at 14,000 rpm for 15 min. The supernatant was taken to dryness using a rotary evaporator at 40 °C. The residue was then reconstituted in 1 mL ACN (80%), filtered through 0.45 μm membrane filter, and injected to the UHPLC-MS/MS system for acquisition of spectra.

### 3.5. Chromatographic and MS Conditions

LC-MS analysis was performed using a Thermo Scientific™ Q Exactive™ Focus Hybrid Quadrupole-Orbitrap™ Mass Spectrometer (Thermo Fisher Scientific, Bremen, Germany) attached to a Thermo Scientific Dionex Ultimate 3000 Series RS pump coupled with a Thermo Scientific Dionex Ultimate 3000 Series TCC-3000RS column compartments and a Thermo Fisher Scientific Ultimate 3000 Series WPS-3000RS autosampler (Thermo Fisher Scientific, Sunnyvale, CA, USA) controlled by Xcalibur Software 4.0 (Thermo Fisher Scientific, Waltham, MA, USA). The chromatographic separation was carried out using a Hypersil GOLD C18 column (100 × 2.1 mm, 1.9 μm) (Thermo Fisher Scientific, Waltham, MA, USA) and the mobile phase consisted of water with 0.1% formic acid (solvent A) and acetonitrile with 0.1% formic acid (solvent B). Gradient elution was run at 0.3 mL/min, with 10% solvent B at the start (t = 0 min), increasing to 70% solvent B at t = 5 min. The gradient was then maintained for 3 min at 70% solvent B until t = 8 min before again increasing solvent B to 100% until the end of the run at t = 9.5 min. The temperature of the autosampler was set to 4 °C. For the metabolism study, the same chromatographic conditions were used with a different elution system. A linear gradient elution from 0% to 100% of solvent B for 45 min was used.

Mass spectrometric detection was operated under a heated electrospray ion source (H-ESI II) in negative ionization mode. The H-ESI parameters were optimized as follows: spray voltage 3.5 kV, capillary temperature 320 °C, S-lens RF level 55, sheath gas flow rate 45, auxiliary gas 10 and sweep gas 2 (manufacturer’s units). Nitrogen was used for the source, Orbitrap bath gas, and the higher energy collision dissociation (HCD) cell collision. A targeted SIM data-dependent MS/MS (tSIM/ddMS2) acquisition was used with an inclusion mass list containing *m*/*z* and retention time of tHGA. The resolution was 35,000 (FWHM) at *m*/*z* 200, with automatic gain control (AGC) target of 5 × 10^4^ ions and an auto maximum ion injection time (IT).

For the identification of tHGA and its metabolites simultaneously, MS detection was operated under a heated electrospray ion source (H-ESI II) using positive/negative switching ionization mode to maximize metabolite coverage in every injection. The H-ESI parameters were optimized as follows: spray voltage (+) and (−) 4.2 and 3.5 kV respectively, capillary temperature 320 °C, S-lens RF level 55, sheath gas flow rate 45, auxiliary gas 10 and sweep gas 2 (manufacturer’s units). Nitrogen was used for the source, Orbitrap bath gas, and the higher energy collision dissociation (HCD) cell collision. The full-scan MS data were recorded across the mass range of 50–1500 *m*/*z*. The resolution was 70,000 (FWHM) at *m*/*z* 200, with automatic gain control (AGC) target of 1 × 10^6^ ions and an auto maximum ion injection time (IT).

### 3.6. Method Validation

The developed method was validated for rat plasma following the European Medicines Agency guidelines (EMA) [25]. The selectivity was evaluated by any interferents observed at the retention times and mass transitions of tHGA and the IS in six different blank rat plasma samples which were processed using the same sample preparation method and injected into the UHPLC-MS/MS system. The chromatograms of blank plasma samples were compared to that of known concentrations of tHGA plasma samples.

The calibration curve was assessed to evaluate the response of the LC-HRMS instrument with regards to the concentration of tHGA over the range of 0.5–80 ng/mL. A total of seven calibration curve points were prepared using plasma samples spiked with various concentrations of tHGA (0.5, 1, 5, 10, 20, 40 and 80 ng/mL) and with 10 µg/mL of the IS solution. The points were run in duplicates, and the linear regression was expressed as y = ax + b, where y is the peak area of tHGA divided by the peak area of IS; a is the slope of the curve; x is the measured concentration of tHGA and b is the intercept of the curve on the y-axis. The coefficient of determination (R^2^) was used to estimate the linearity of the standard curve.

The lower limit of quantification (LLOQ) was chosen to be the lowest concentration of the standard curve at which the accuracy and precision were within ±20%. LLOQ was established by measuring six spiked plasma samples.

Within-run accuracy and precision were determined using six replicates of QC samples at four different concentrations (0.5, 1.5, 35, and 70 ng/mL). Between-run accuracy and precision were determined using six replicates of the QC samples at four concentrations in three different days. The accuracy and the precision were expressed by percent relative error (RE) and relative standard deviation (RSD), respectively. RE and RSD should be less than 15% except for LLOQ less than 20%.

To assess the matrix effect, six blank plasma samples were extracted using PPT then spiked with tHGA at three QC concentration levels (1.5, 35 and 70 ng/mL). In addition, six samples of tHGA were dissolved in the solvent (80% acetonitrile in water) at equivalent concentrations. The matrix effect was calculated by comparing the peak areas of post-extraction spiked samples with the peak areas of tHGA in the solvent and expressed as a percentage. The effect should be ≤15%.

The recovery percentage was measured by comparing the peak area of six replicates of QC samples (pre-extraction) at three QC levels (1.5, 35 and 70 ng/mL) with the peak area of six post-extraction spiked samples at equivalent concentrations. The percent recovery was calculated and expressed as a percentage.

A dilution integrity test was conducted using a high concentration sample of tHGA prepared in rat plasma (500 ng/mL) which was about six times beyond the upper limit of quantification (80 ng/mL). The analysis was demonstrated after a 10-fold dilution of the concentrated sample with blank plasma to reach a concentration of 50 ng/mL. The mean accuracy after dilution, expressed by RE, and precision, expressed by RSD should be ≤15%.

Carry-over was evaluated by injecting blank plasma samples immediately after the upper limit of quantification in duplicate and the response of the blank plasma was compared with the response of the lower limit of quantification. The ratio should be ≤20%.

Stability tests were performed to ensure that all steps in sample preparation, analysis, and storage did not affect the concentration of tHGA. Short- and long-term stability studies of tHGA in plasma was assessed using six replicates at two concentration levels, LQC and HQC. The QC samples were analyzed against the standard curve which was freshly prepared. The short-term stability was evaluated by analyzing the processed QC samples kept in the autosampler (4 °C) for 24 h. For long-term stability, tHGA plasma samples in low and high concentrations were stored at −80 °C for 1 month.

### 3.7. Data Analysis

The pharmacokinetic parameters namely, maximum concentration (C_max_), the elimination half-life (t_1/2_) area under the curve (AUC_0–24_) were analyzed using ThothPro software (ThothPro™, version 4.3.0, Gdansk, Poland) after intraperitoneal and oral administrations at a dose of 20 mg/kg of tHGA by non-compartmental model and analyzed using Statistical Package for the Social Science Version 20.0 (SPSS Inc. Chicago, IL, USA). AUC_0–t_ was obtained by a linear-up/log-down trapezoidal method. The time to reach maximum concentration (t_max_) was obtained from a concentration-time curve, the relative bioavailability was calculated according to the following equation:

F_R_ = (AUC_PO_ × D_IP_)/(AUC_IP_ × D_PO_) × 100 (1)

where F_R_ is the relative bioavailability; AUC_PO_ is the area under the curve of a plasma concentration versus time plot for PO administration; D_O_ is the amount of drug dosed by PO administration; AUC_IP_ is the area under the curve of a plasma concentration versus time plot for intraperitoneal administration; D_IP_ is the amount of drug dosed by intraperitoneal administration.

All MS data acquired using the Q Exactive MS were controlled by Xcalibur 4.0 software (Thermo Fisher Scientific). The MS data from plasma-, urine-, and feces-treated, as well as the control samples were analyzed using Compound Discoverer software 2.1 (Thermo Fisher Scientific) for the identification of tHGA metabolites. The software extracted information about metabolite *m*/*z* values depending on a list of expected metabolites. As no standards or literature information were available to confirm tHGA metabolites structure, tentative elucidation of the metabolites was performed according to Schymanski classification [26]. A list of putative metabolites with the probable metabolism pathway was created for peaks based on MS, MS/MS fragmentation and the isotopic pattern of tHGA. The matching percentage between measured and theoretical MS/MS spectra was determined depending on the existence/absence of theoretical fragments in the MS/MS spectra generated experimentally. The important parameters of the software are listed in Table 5. In addition, the site of metabolism was determined based on the tHGA structure with the help of a web server site for metabolism predictor [27,28].

## 4. Conclusions

In summary, an LC-HRMS method to quantify tHGA in rat plasma was developed and validated. The developed method was stable, accurate, and precise with a high-level of sensitivity and selectivity which makes it ideal for determining tHGA concentrations in complex matrices such as blood and plasma. The method was successfully utilized to evaluate the pharmacokinetics and bioavailability of free and encapsulated tHGA after oral and intraperitoneal drug administrations. The results showed improvement in the pharmacokinetic profile of the liposomal formulation when compared with free tHGA. The pharmacokinetic profile of tHGA after intraperitoneal administration was significantly better than the oral one. C_max_ of IP administration was 3.8-fold and 10.2-fold greater than C_max_ of encapsulated and free tHGA, respectively. The relative oral bioavailability of unformulated and encapsulated tHGA was 9.1 and 21.0%, respectively. The relative oral bioavailability of the liposomal formulation of tHGA increased by 2.3-fold compared to the free tHGA. Additionally, the metabolism of tHGA was studied using high-resolution orbitrap MS. A total of twelve metabolites were detected and identified in plasma, urine, and feces samples. The phase-I metabolic transformations of tHGA were found to be hydroxylation, dihydroxylation, desaturation, and hydrolysis at the geranyl substituent, while phase-II metabolic transformations were mainly glucuronidation and one sulfation metabolite on the hydroxyl group of the aromatic ring. The results of the present study have provided additional insights into the drug-like properties of tHGA which will be useful for further studies into its potential use as a therapeutic drug.

## Figures and Tables

**Table 1:** Within- and between run accuracies and precisions for tHGA determination in rat plasma at LLOQ, low quality control (LQC), medium quality control (MQC), and high quality control (n = 6).

tHGA (ng/mL)	Within-Run		Between-Run (3 days)		Range %
	Accuracy RE %	Precision RSD %	Accuracy RE %	Precision RSD %	
0.5	9.4	6.8	7.2	6.9	≤20
1.5	8.6	4.2	−2.9	8.7	≤15
35	−4.1	6.0	3.1	8.5	≤15
70	−7.8	5.1	−1.3	6.7	≤15

**Table 2:** The matrix effect, recovery, and stability of tHGA in rat plasma (n = 6).

tHGA (ng/mL)	ME (%)	Recovery tHGA (%)	Recovery IS (%)	Stability RE (%)	
				Short-Term (4 °C)	Long-Term (−80 °C)
1.5	91.4 ± 4.2	99.6 ± 11.7	99.6 ± 2.8	−4.66 ± 11.9	5.93 ± 2.8
35	104.1 ± 6.1	100.3 ± 10.5	99.6 ± 3.5	-	-
70	107.8 ± 5.1	96.3 ± 11.0	95.6 ± 3.3	5.34 ± 6.9	5.34 ± 5.1

**Table 3:** Pharmacokinetics parameters and relative oral bioavailability of tHGA following PO administration of the free and liposomal formulation of tHGA and IP administration of tHGA at the dose of 20 mg/kg.

Parameter	Unit	PO		IP tHGA-Loaded Liposomes
		Free tHGA	tHGA-Loaded Liposomes	
C_max_	ng/mL	5.35	14.54	54.6
t_max_	h	0.25	0.25	1.5
t_1/2_	h	6.95	6.65	6.67
AUC_0-24_	ng·h/mL	17.60	40.72	193.90
F_r_	%	9.07	21.00	

Data are represented as the mean value (n = 6). AUC_0__–__24_ is the area under the curve from time zero to the last measured at 24 h post-dose; C_max_ is the maximum concentration; t_max_ is time to achieve maximum concentration; t_1/2_ is elimination half-life; F_r_ is relative bioavailability.

**Table 4:** Mass spectral characterization of tHGA and its metabolites in rat blood, urine and feces.

ID	Pathway	*m*/*z*	RT	Formula	Nominal MW	Measured MW	Δ Mass (ppm)	MS2	Source *
M0		[M − H] − 303.1602	25.7	C_18_H_24_O_4_	304.1675	304.1673	0.2	303.1602, 261.1489, 259.1696, 233.0816, 219.0654, 217.1595, 191.0336, 179.0337, 177.1279, 175.0759, 166.0259, 164.0466, 151.0029, 137.0231, 123.0437, 83.0123, 81.0332, 57.0331	
		[M − H] + 305.1759	25.7	C_18_H_24_O_4_	304.1675	304.1687	1.2	270.7504, 195.0657, 182.0537, 181.0503, 177.0561, 171.3326, 163.0396, 139.0396, 135.0448, 121.0292, 107.0497, 95.0498, 93.0340, 69.0706, 67.0186	
M1	Glucuronide Conjugation	[M − H] − 479.1918	20.5	C_24_H_32_O_10_	480.1996	480.1991	−0.53	303.1601, 261.1500, 259.1700, 179.0343, 166.0261, 123.0439, 113.0231, 99.0075, 95.0126, 85.028, 59.0124	B, U
		[M − H] + 481.2091	20.5	C_24_H_32_O_10_	480.1996	480.1991	2.22	477.5425, 305.1754, 195.0661, 182.0536, 181.0503, 163.0392, 111.1179, 60.7949.	
M2	Hydroxylation (Oxidation)	[M − H] − 319.1543	18.9	C_18_H_24_O_5_	320.1624	320.1614	−0.95	319.1534, 233.0812, 277.1428, 219.0651, 205.0499, 192.0413, 191.0338, 179.0337, 166.0258, 164.0470, 151.0021, 137.0231, 123.0438, 83.0124, 81.0331, 57.0331.	F, U
M3	Hydroxylation (Oxidation)	[M − H] − 319.1543	21.2	C_18_H_24_ O_5_	320.1624	320.1614	−0.95	319.1540, 261.1131, 232.0728, 217.0499, 192.0411, 179.0338, 177.0180, 163.0023, 151.0388, 149.0596, 137.0231, 109.0283, 107.0488, 83.0123.	F, U
M4	Dihydroxylation	[M − H] − 335.1493	19.0	C_18_H_24_O_6_	336.1572	336.1564	−0.93	293.1387, 249.1486, 225.1484, 209.1178, 207.1379, 205.1585, 166.0259, 165.1267, 137.0961, 125.0230, 124.0148, 123.0438, 109.0643, 97.0644, 83.0123, 81.0331, 73.0281, 57.0332	F
		[M − H] + 337.1659	19.0	C_18_H_24_O_6_	336.1572	336.1585	1.24	277.1446, 259.1332, 181.0503, 163.0396, 139.0397, 135.0444, 121.0289, 67.0187	
M5	Hydrolysis	[M − H] − 321.1700	19.8	C_18_H_26_O_5_	322.1780	322.1771	0.07	279.1595, 277.1806, 237.1493, 235.1697, 219.1384, 192.0419, 191.0343, 179.0339, 166.0260, 164.0462, 124.0152, 123.0439, 122.0362, 83.0124, 81.0332, 57.0332	F
		[M − H] + 323.1867	19.8	C_18_H_26_O_5_	322.1780	322.1796	2.45	Not detected	
M6	Desaturation of M4	[M − H] − 333.1337	19.0	C_18_H_22_O_6_	334.1416	334.1410	−0.66	247.1330, 233.0818, 223.1329, 205.1226, 203.1433, 191.0341, 179.0339, 166.0260, 163.1116, 138.0309, 125.0233, 124.0152, 123.0441, 109.0645, 99.0438, 83.0123, 81.0332, 57.0332	F, B
M7	Desaturation	[M − H] − 301.1438	25.4	C_18_H_22_O_4_	302.1518	302.1509	−0.92	257.1542, 232.0729, 231.0654, 217.0497, 204.0775, 192.0415, 191.0340, 187.0754, 179.0337, 166.0258, 164.0464, 156.0053, 152.0101, 124.0151, 85.0281, 83.0123	F
M8	Glucuronidation of M2	[M − H] − 495.1870	15.4	C_18_H_24_O_4_	496.1945	496.1948	0.07	361.1650, 319.1548, 277.1439, 275.1522, 179.0338, 166.0258, 123.0440, 113.0233, 99.0074, 95.0123, 85.0281, 71.0125, 59.0125	U, B
		[M − H] + 497.2041	15.4	C_24_H_32_O_11_	496.1945	496.1948	2.45	Not detected	
M9	Sulfation of M4	[M − H] − 415.1066	15.8	C_18_H_24_O_9_S	416.1141	416.1138	0.07	336.1529, 335.1499, 293.1394, 291.1600, 273.1282, 251.1282, 249.1493, 209.1174, 207.1384, 205.1593, 165.1277, 123.0440, 83.0126, 81.0332, 57.0332	B
M10	Hydroxylation of M5	[M − H] − 337.1650	15.5 16.8	C_18_H_26_O_6_	338.1729	338.1722	−0.74	295.1546, 251.1645, 192.0416, 191.0335, 179.0333, 177.1271, 166.0261, 164.0466, 123.0438, 83.0123, 81.0331, 73.0280	F
M11	Glucuronidation of M5	[M − H] − 497.2022	17.7	C_24_H_34_O_11_	498.2101	498.2095	−0.63	387.1804, 363.1807, 321.1703, 279.1595, 277.1802, 151.0750, 113.0228, 85.0281, 83.0125, 81.0332, 59.0125	U, B
M12	Glucuronidation of M4	[M − H] − 511.1813	15.8	C_24_H_32_O_12_	512.1894	512.1887	−0.67	335.1496, 293.1391, 291.1599, 251.1280, 249.1501, 247.1691, 207.1382, 205.1588, 175.0238, 113.0230, 99.0074, 95.0124, 85.0280, 59.0123, 57.0331	U, B
		[M − H] + 513.1987	15.8		512.1894	512.19176	2.38	Not detected	

* B, U and F represent blood, urine and feces samples, respectively.

**Table 5:** Compound discoverer software parameters.

**Expected Compound Generator**	
Parent compound	tHGA (C_18_H_24_O_4_)
Phase-I metabolism	Dehydration (−H_2_O), desaturation (−H_2_), hydration (+H_2_O), oxidation (+O), reduction (−H_2_).
Phase-II metabolism	Acetylation, arginine conjugation, Cysteine conjugation, glucoside conjugation, glucuronide conjugation, glutamine conjugation, glycine conjugation, GSH conjugation (on bromine), GSH conjugation 1, GSH conjugation 2, methylation, ornithine conjugation, palmitoyl conjugation, stearyl conjugation, sulfation, taurine conjugation
Maximum numbers of phase-II reactions	1
Maximum numbers of reactions	3
Ionisation	[M + H]^+^, [M + K]^+^, [M + Na]^+^, [M − H]^−^
**Expected Compounds Finder**	
Mass tolerance	5 ppm
Intensity tolerance	30
Intensity threshold	0.1
Sn threshold	3
Minimum isotopes	2
Minimum peak intensity	10,000
**Group Expected Compounds**	
Rt tolerance	0.1 min
Fragments data selection (preferred ions)	[M + H]^+^, [M − H]^−^
**Fish Scoring**	
S/n threshold	20
High accuracy mass Tolerance	2.5 mmu
Low accuracy mass Tolerance	0.5 Da
Maximum steps in the fragmentation pathway	5
Minimum fragment	50

## Supplementary Materials

Supplementary materials are available online.
