# Supplementary material for: Pharmacokinetics and Metabolism of Liposome-Encapsulated 2,4,6-Trihydroxygeranylacetophenone in Rats Using High-Resolution Orbitrap Liquid Chromatography Mass Spectrometry
Source: Molecules. 2020 Jul 6;25(13):3069. doi: 10.3390/molecules25133069 (PMC7412073; doi:10.3390/molecules25133069)
Supplement: Supplementary file 1 [file molecules-25-03069-s001.pdf]

Pharmacokinetics and Metabolism of Liposome-Encapsulated 2,4,6-Trihydroxygeranylacetophenone in Rats Using High-Resolution Orbitrap Liquid Chromatography Mass Spectrometry

Yamen Alkhateeb [1], Qais Bashir Jarrar [2], Faridah Abas [3], Yaya Rukayadi [3], Chau Ling Tham [4], Yuen Kah Hay [5] and Khozirah Shaari [1,3,*]

1 Laboratory of Natural Product, Institute of Bioscience, Universiti Putra Malaysia, 43400, Serdang, Selangor, Malaysia; yamen.alkhatib84@gmail.com
2 Department of Applied Pharmaceutical Sciences, Faculty of Pharmacy, Al-Isra University, 11622 Amman, Jordan; jarrarq@yahoo.com
3 Department of Food Science, Faculty of Food Science and Technology, Universiti Putra Malaysia, 43400, Serdang, Selangor, Malaysia; faridah_abas@upm.edu.my (F.A.); yaya_rukayadi@upm.edu.my (Y.R.)
4 Department of Biomedical Science, Faculty of Medicine & Health Sciences, Universiti Putra Malaysia, 43400 UPM Serdang, Selangor, Malaysia; chauling@upm.edu.my
5 School of Pharmaceutical Sciences, Universiti Sains Malaysia, 11800, Glugor, Penang, Malaysia; khyuen@usm.my
* Correspondence: khozirah@upm.edu.my; Tel.: +603-9769-1481

Academic Editor: Young G. Shin

Received: 30 April 2020; Accepted: 20 May 2020; Published: date

Preparation of tHGA

The compound was prepared according to a described method (Ismail et al. 2012) via the reaction of phloracetophenone (1.000 g, 6 mmol), geranyl bromide (0.876 g, 4.80 mmol), and anhydrous potassium carbonate (0.415 g, 3.00 mmol) in dry acetone (3.5 mL). The mixture was stirred well and then refluxed for 6 h. The reaction mixture was filtered and evaporated under reduced pressure.

Preparation and Characterization of Liposomes-Encapsulated tHGA

The liposomal formulation of tHGA was prepared from proliposomes (contained 50% unsaturated soybean phosphatidylcholines suspended in a specific quantity of food-grade hydrophilic medium) (Prolipo™ Duo) following the manufacturer's instructions with adjustments made to the dilution steps. Stock solution (128 mg/mL) of tHGA was prepared by dissolving tHGA in DMSO. Then, 250 µL of the stock solution was slowly added into 1 g of proliposomes with stirring. Next, the mixture was hydrated by adding 2 mL of distilled water gradually with stirring for 1 h. The mixture finally diluted with water to reach a total volume of 8 mL. The blank liposomal formulation was prepared using the same steps with no addition of tHGA.

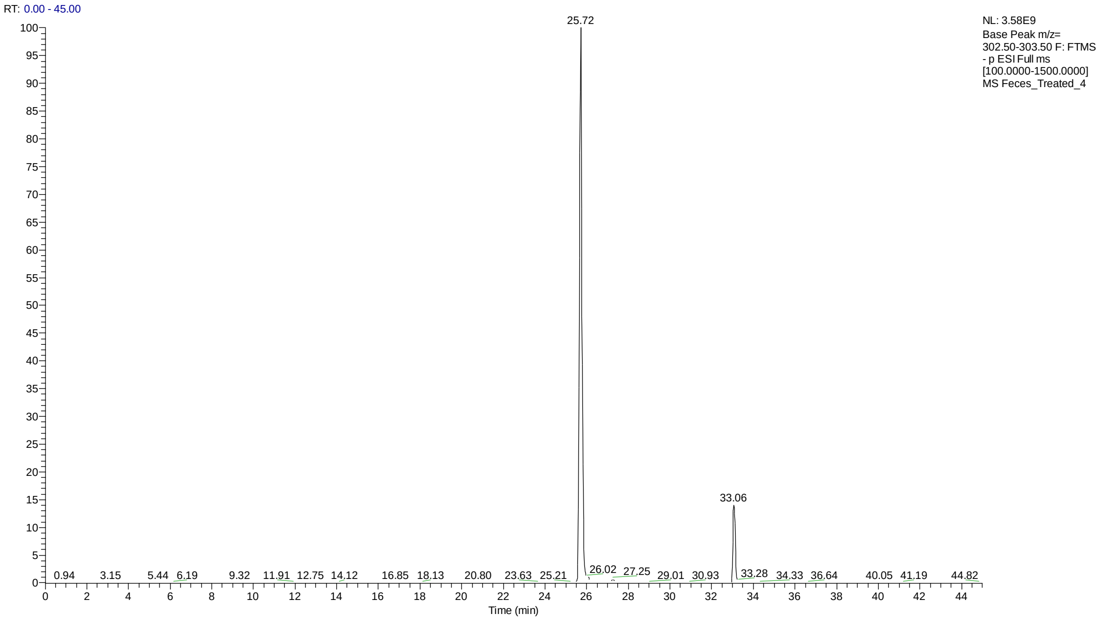

Figure S1. LC-MS chromatogram of tHGA.

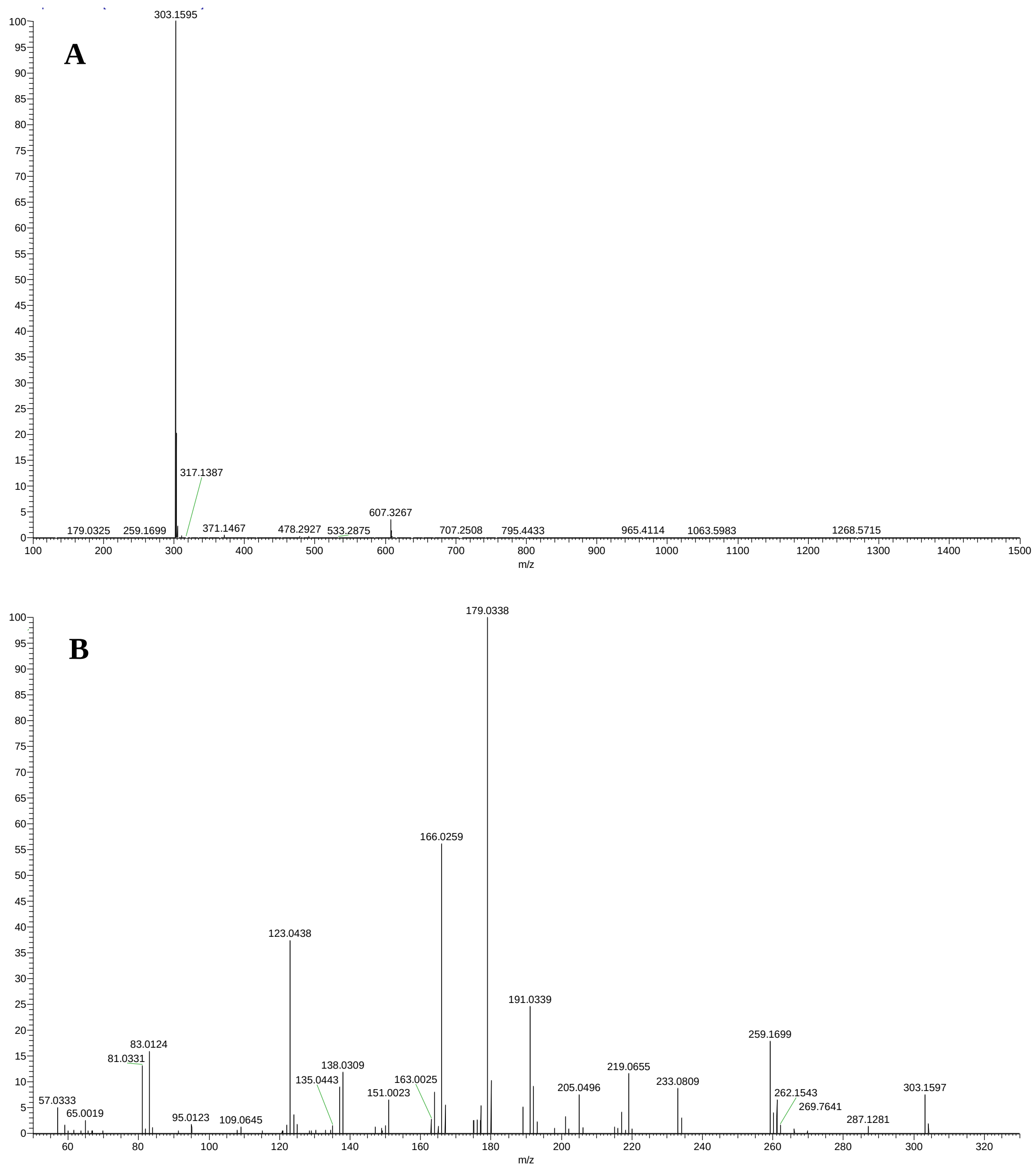

Figure S2. Characterisation of tHGA in negative ion mode, (**A**) MS spectrum and (**B**) MS/MS fragmentation spectrum.

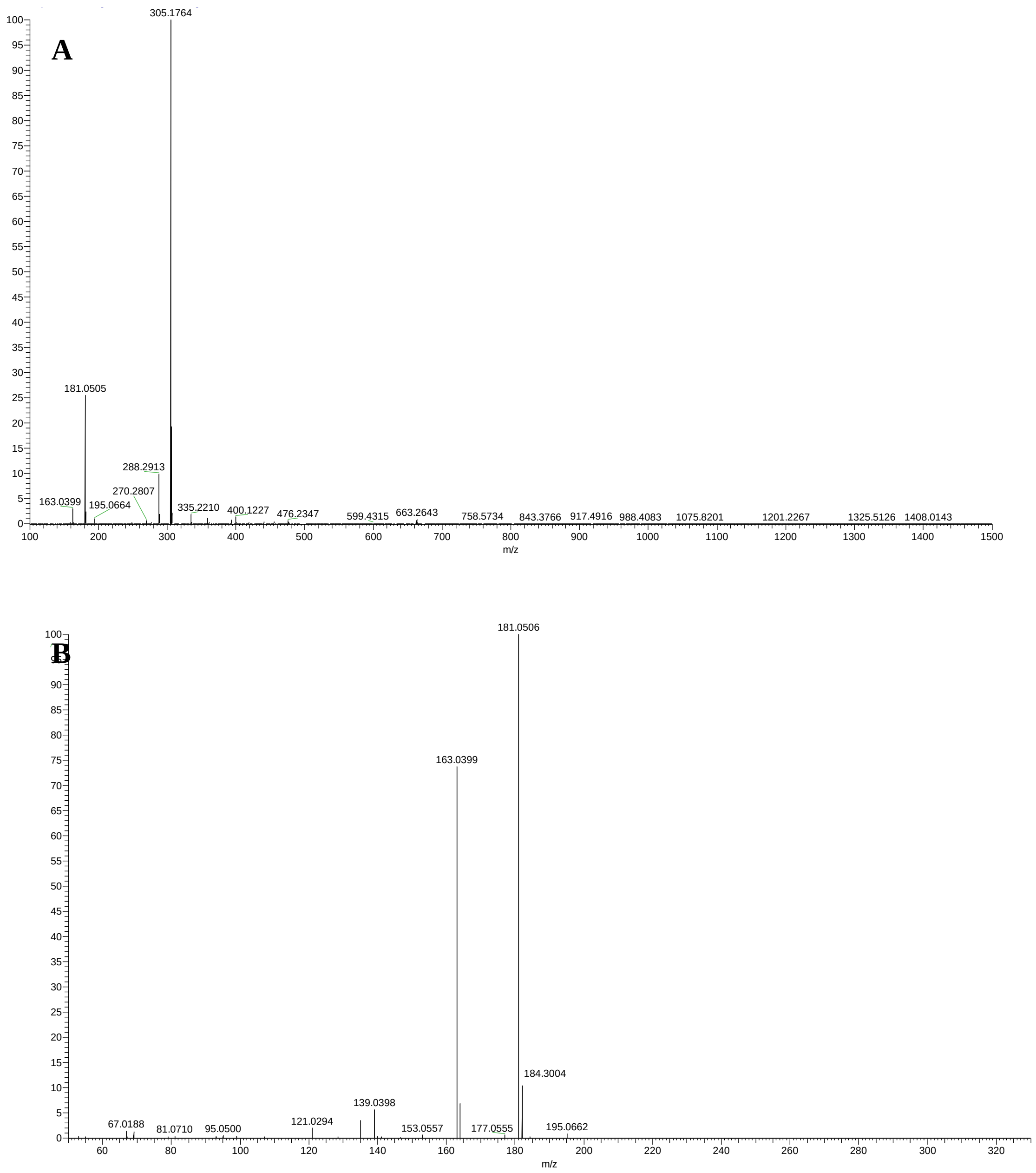

Figure S3. Characterisation of tHGA in positive ion mode, (A) MS spectrum and (B) MS/MS fragmentation spectrum.

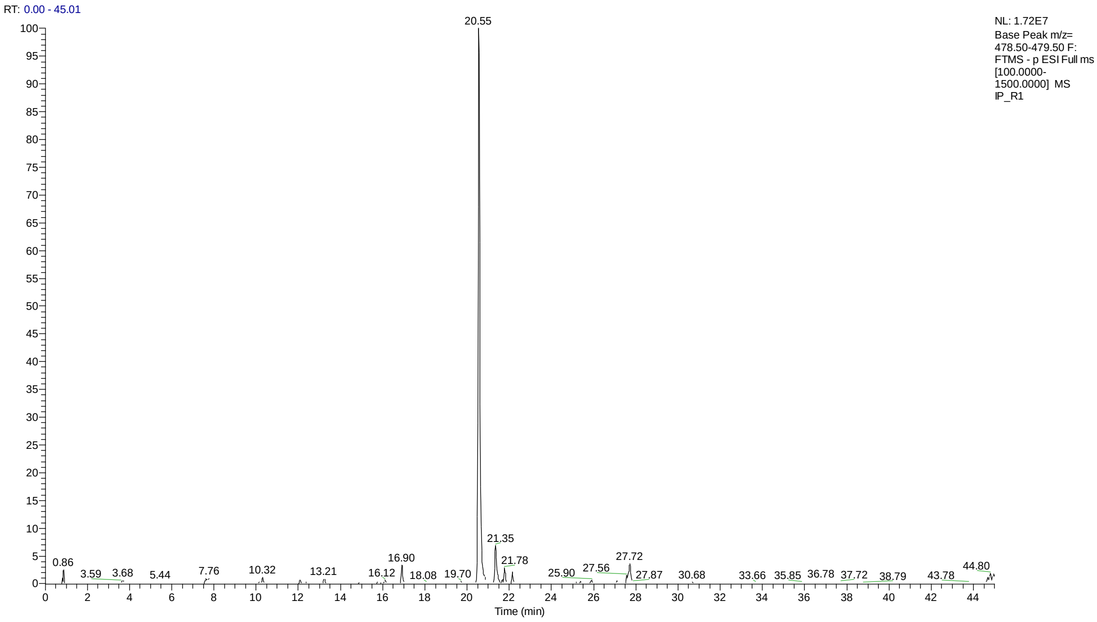

Figure S4. LC-MS chromatogram of the metabolite M1.

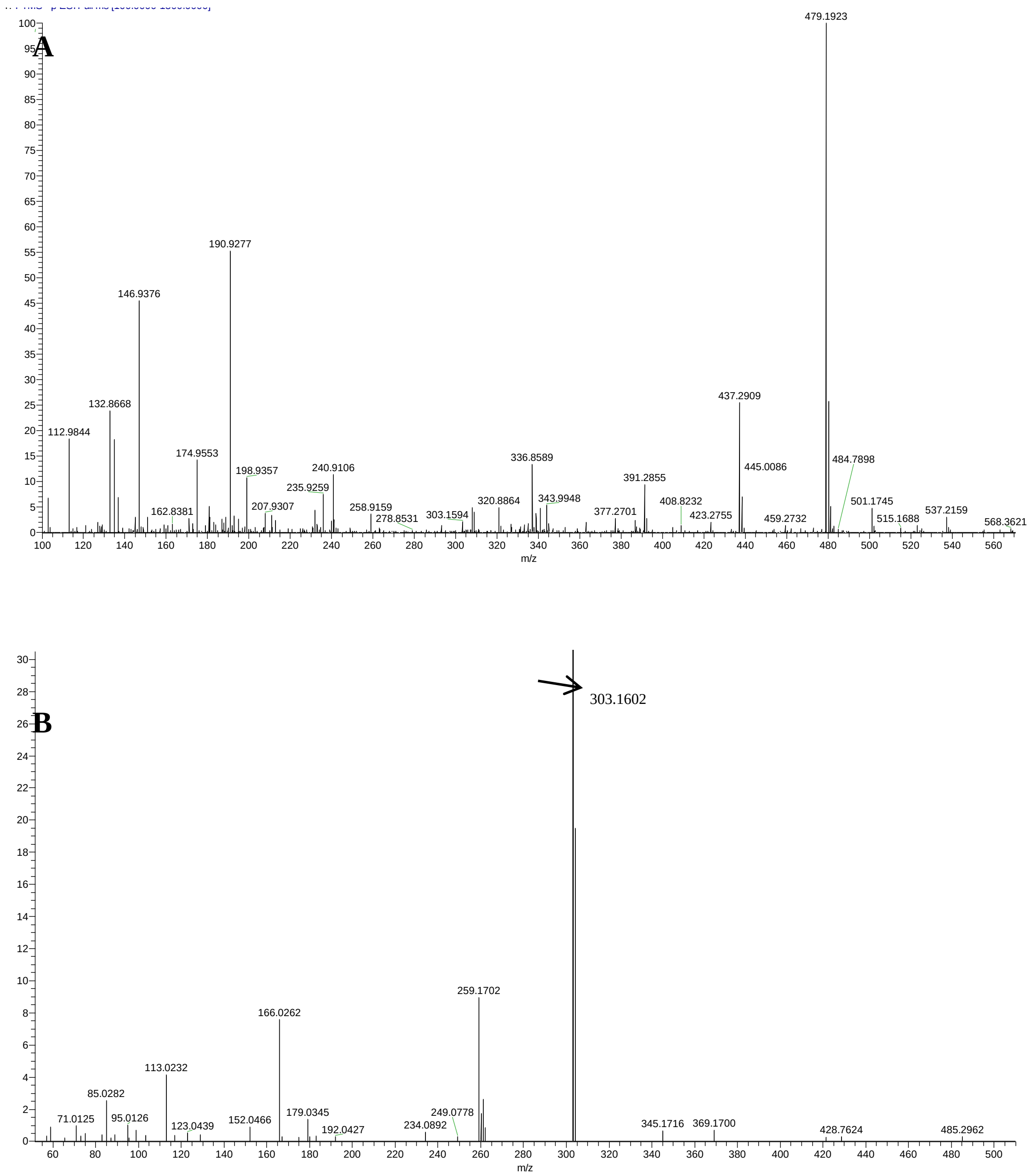

Figure S5. Characterisation of M1 in negative ion mode, (**A**) MS spectrum and (**B**) MS/MS fragmentation spectrum, the intensity was zoomed in range 0–30% due to the very high intensity of the fragment 303.1602.

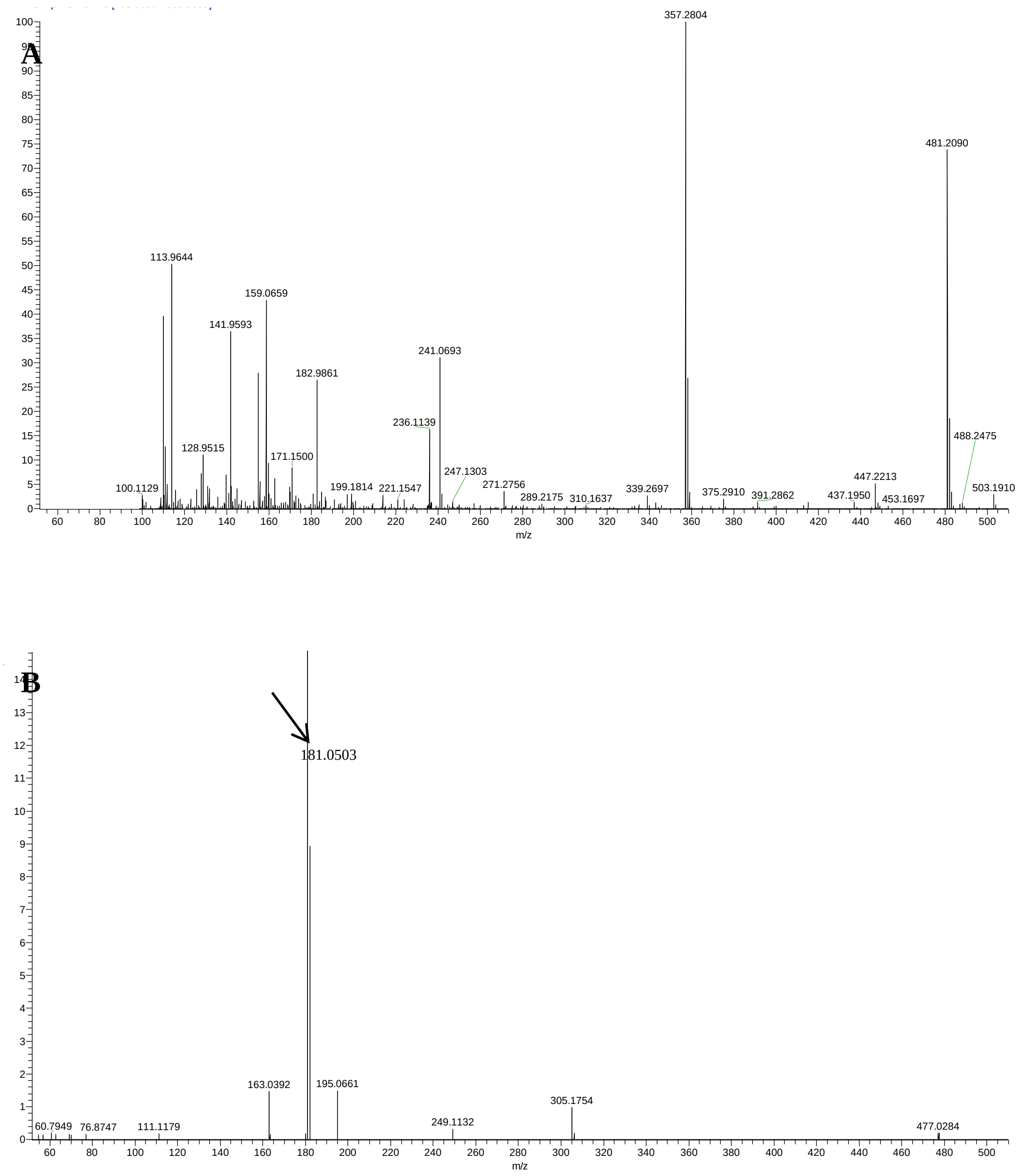

Figure S6. Characterisation of M1 in positive ion mode, (A) MS spectrum and (B) MS/MS fragmentation spectrum, the intensity was zoomed in range 0–15% due to the very high intensity of the fragment 181.0503.

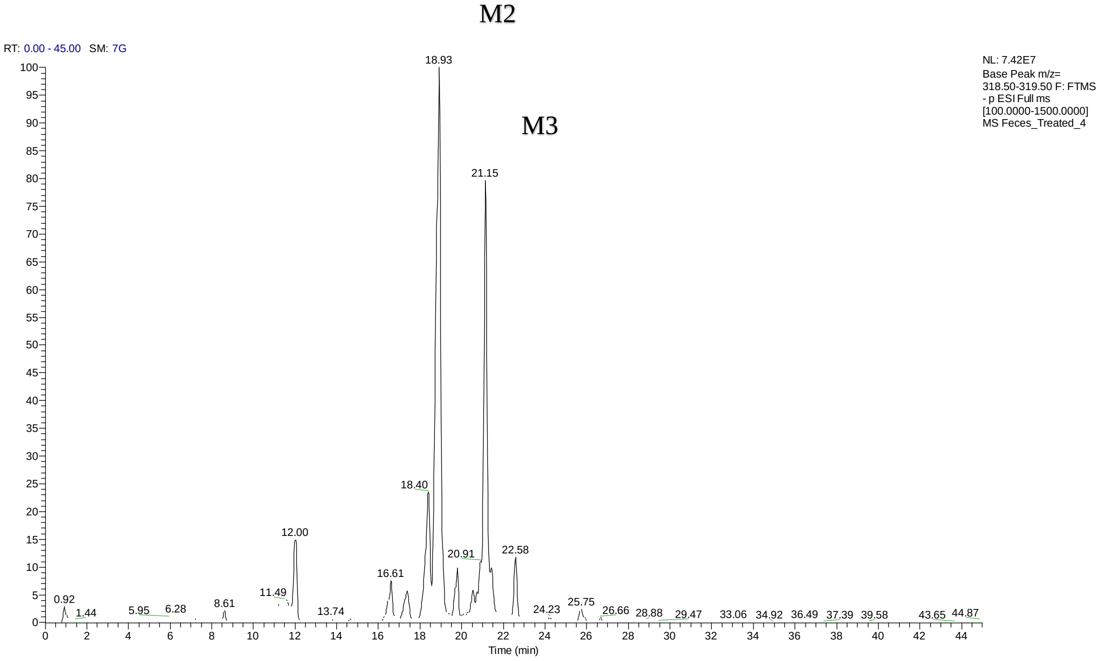

Figure S7. LC-MS chromatogram of the metabolite M2 and M3.

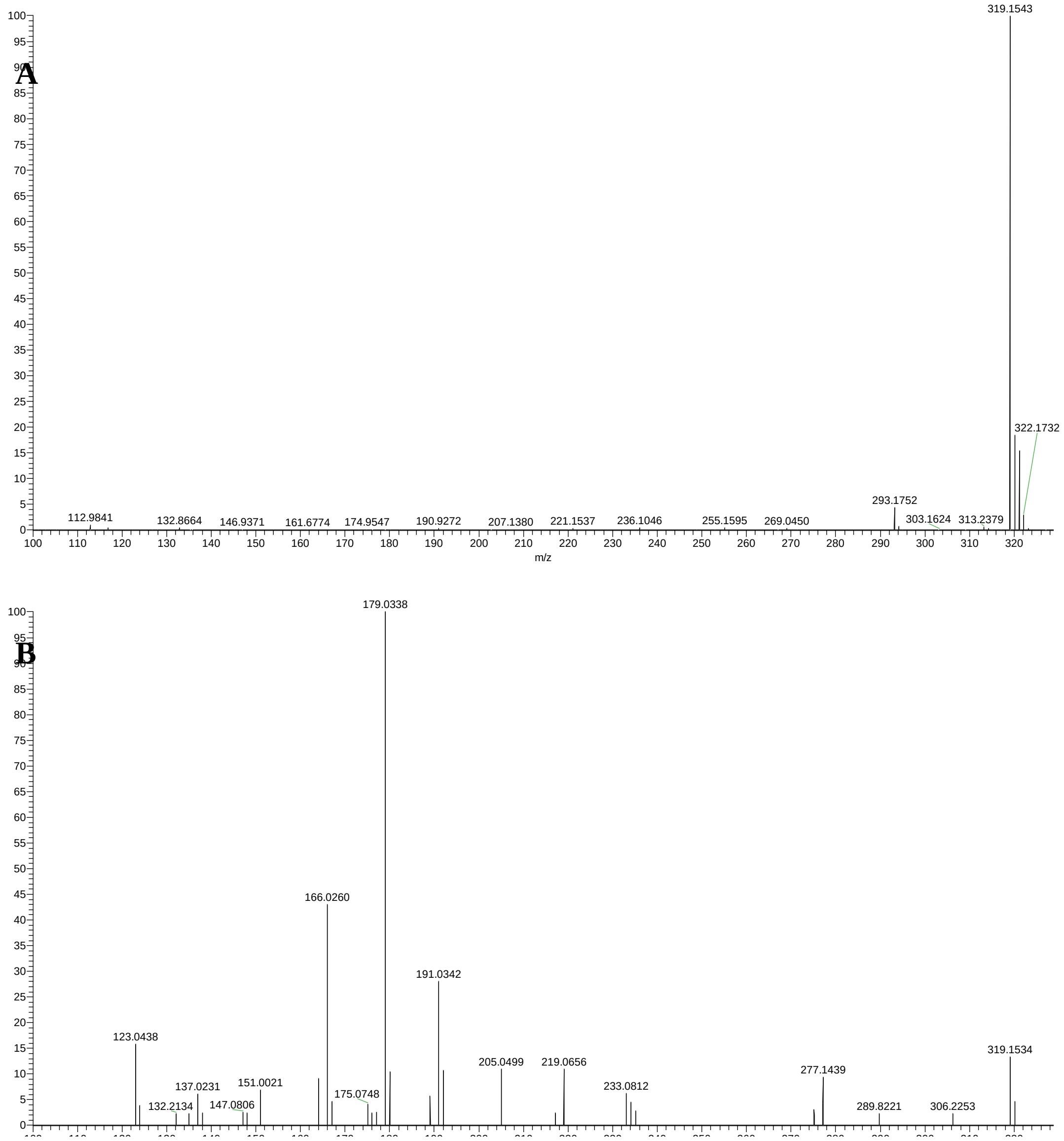

Figure S8. Characterisation of M2 in negative ion mode, (**A**) MS spectrum and (**B**) MS/MS fragmentation spectrum.

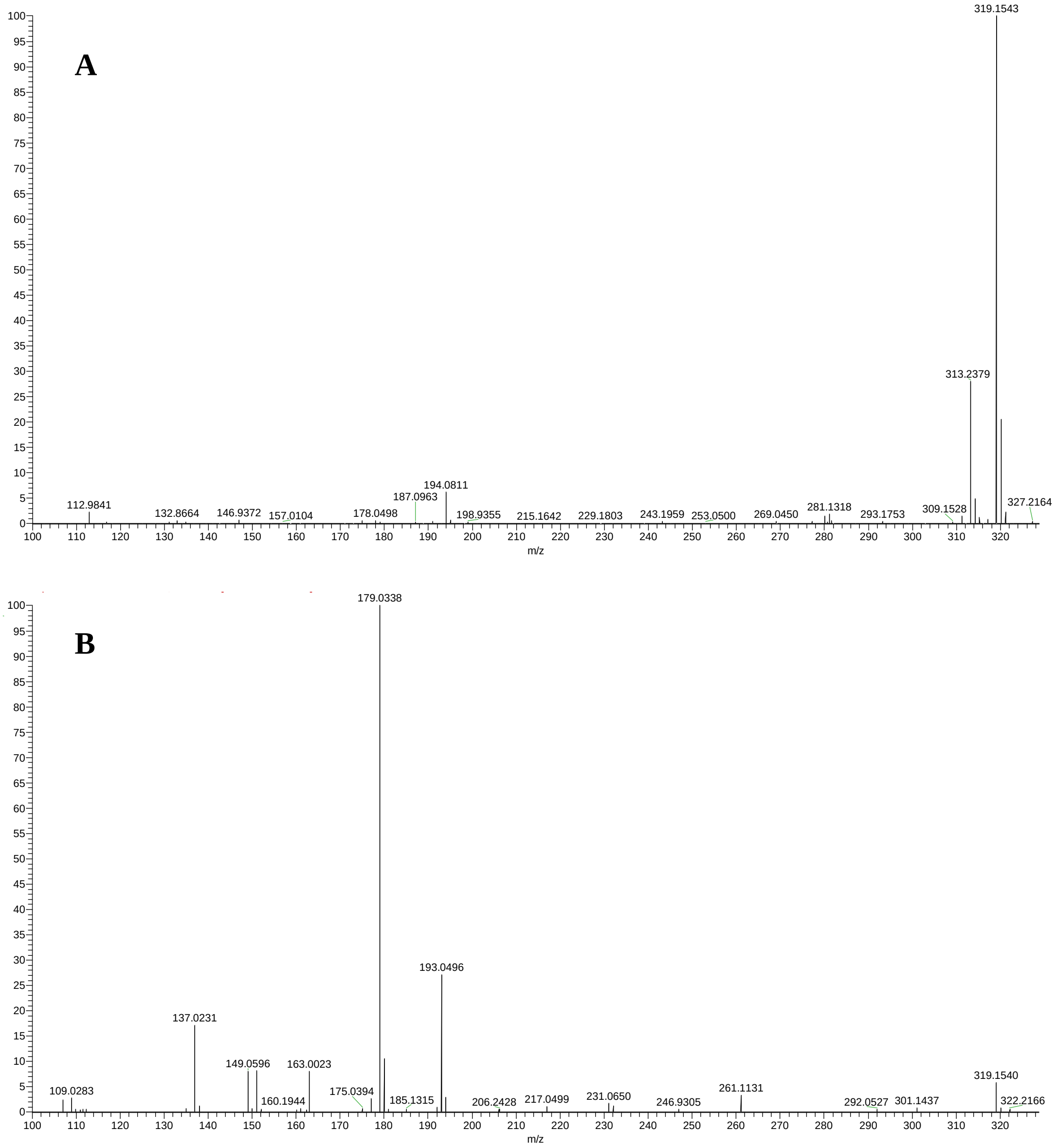

Figure S9. Characterisation of M3 in negative ion mode, (**A**) MS spectrum and (**B**) MS/MS fragmentation spectrum.

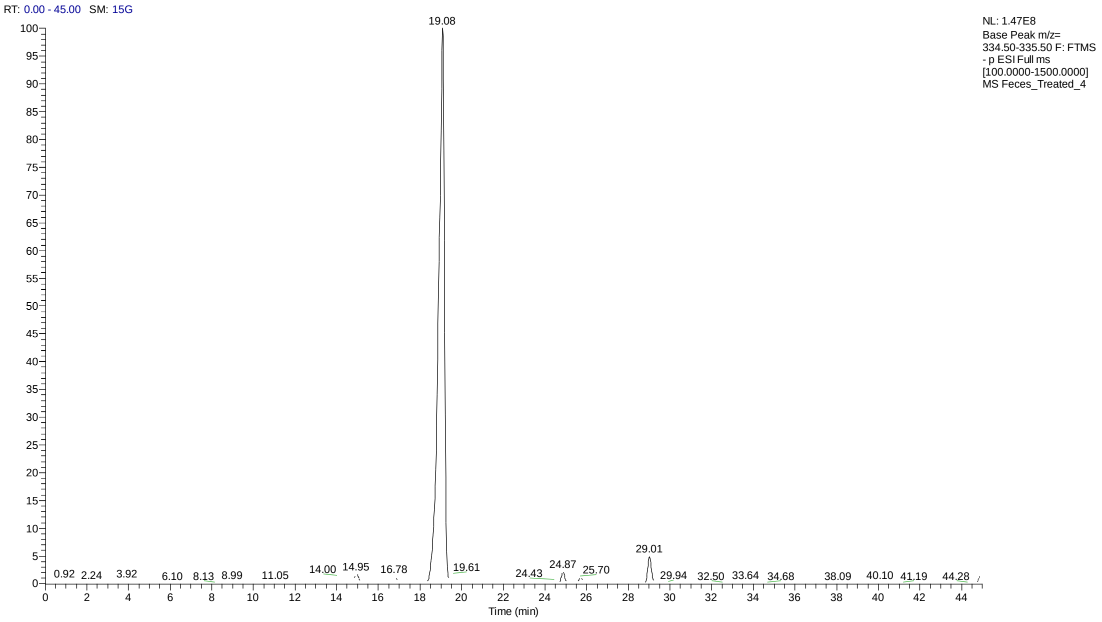

Figure S10. LC-MS chromatogram of the metabolite M4.

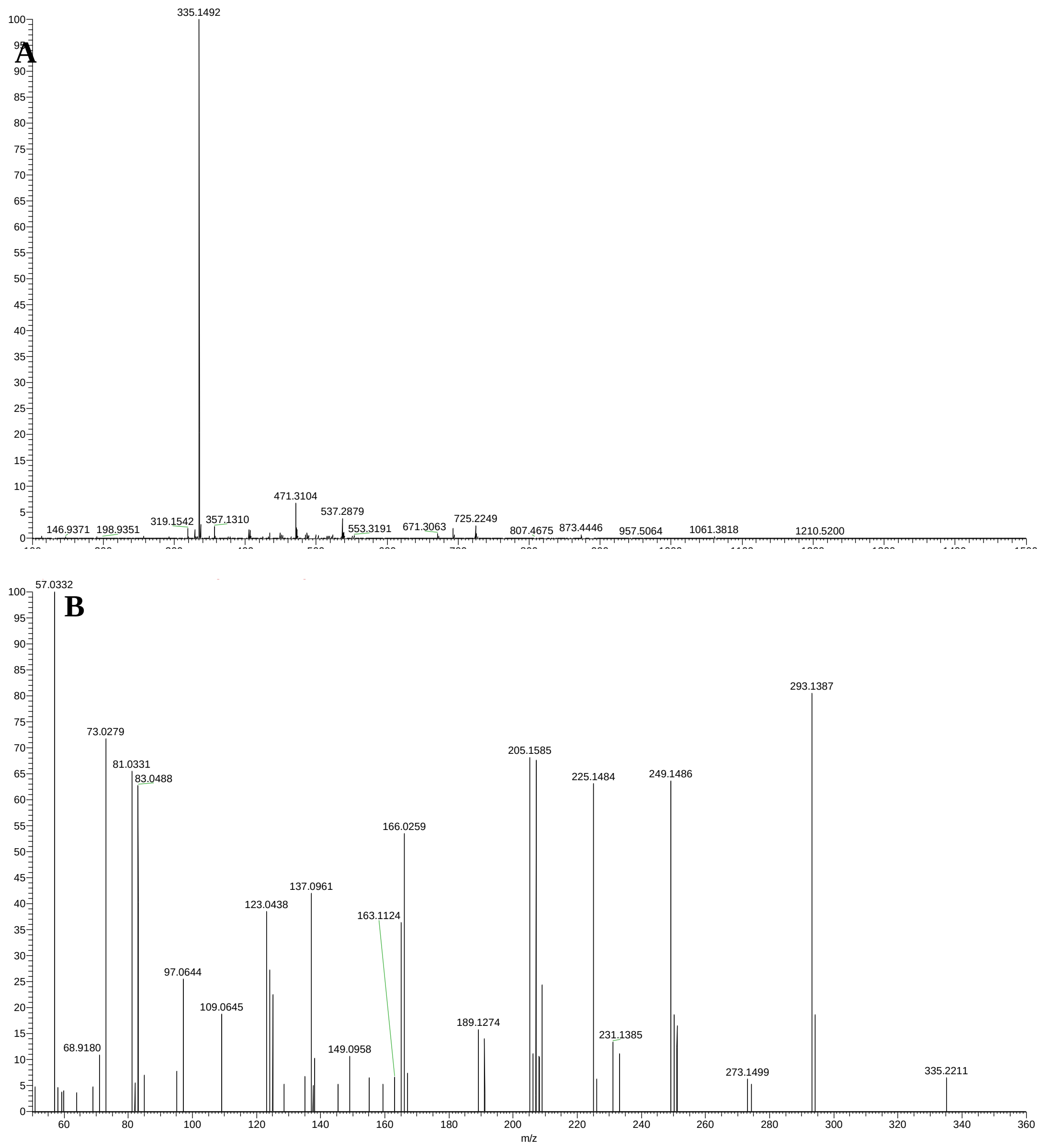

Figure S11. Characterisation of M4 in negative ion mode, (**A**) MS spectrum and (**B**) MS/MS fragmentation spectrum.

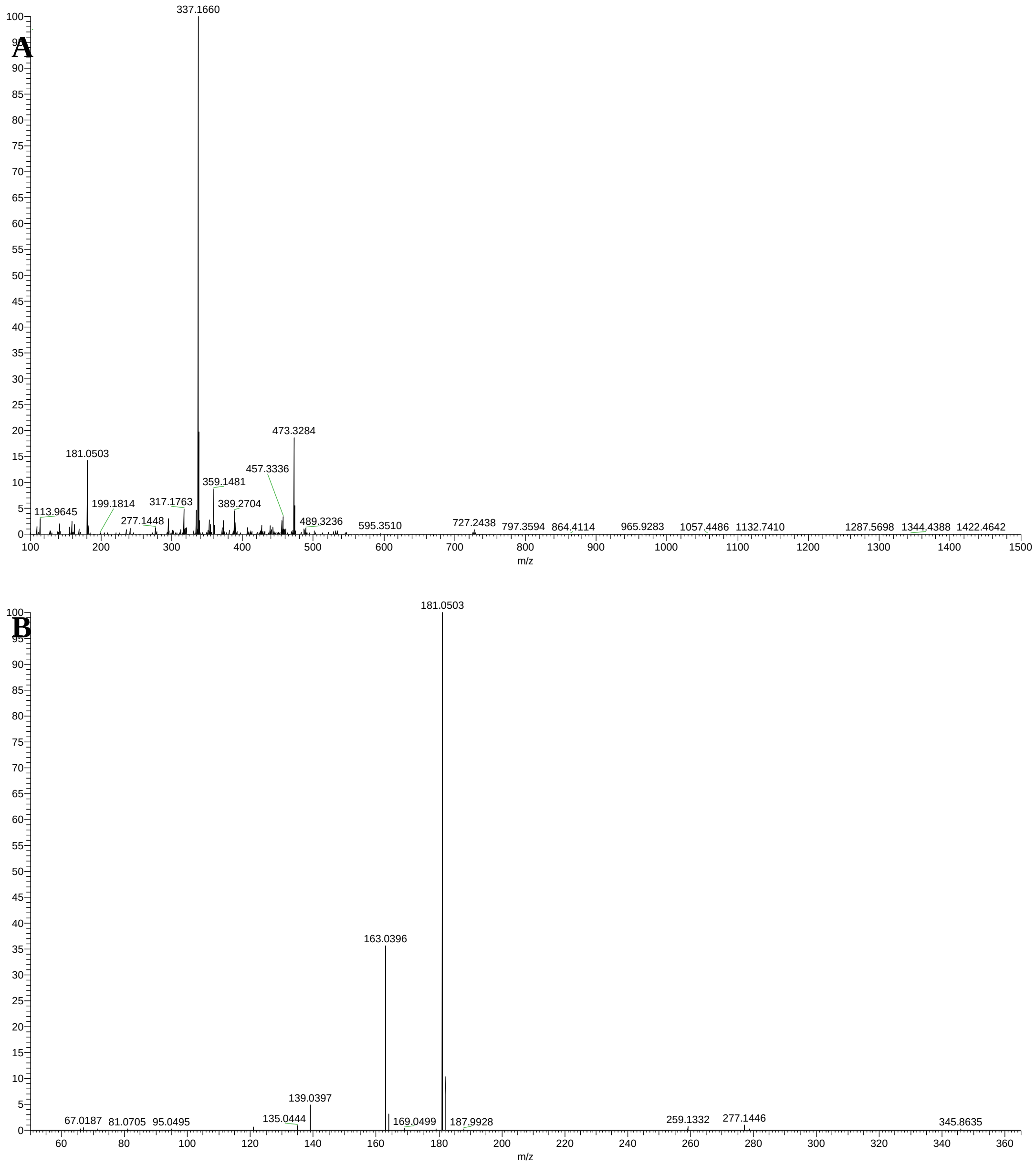

Figure S12. Characterisation of M4 in positive ion mode, (**A**) MS spectrum and (**B**) MS/MS fragmentation spectrum.

M5

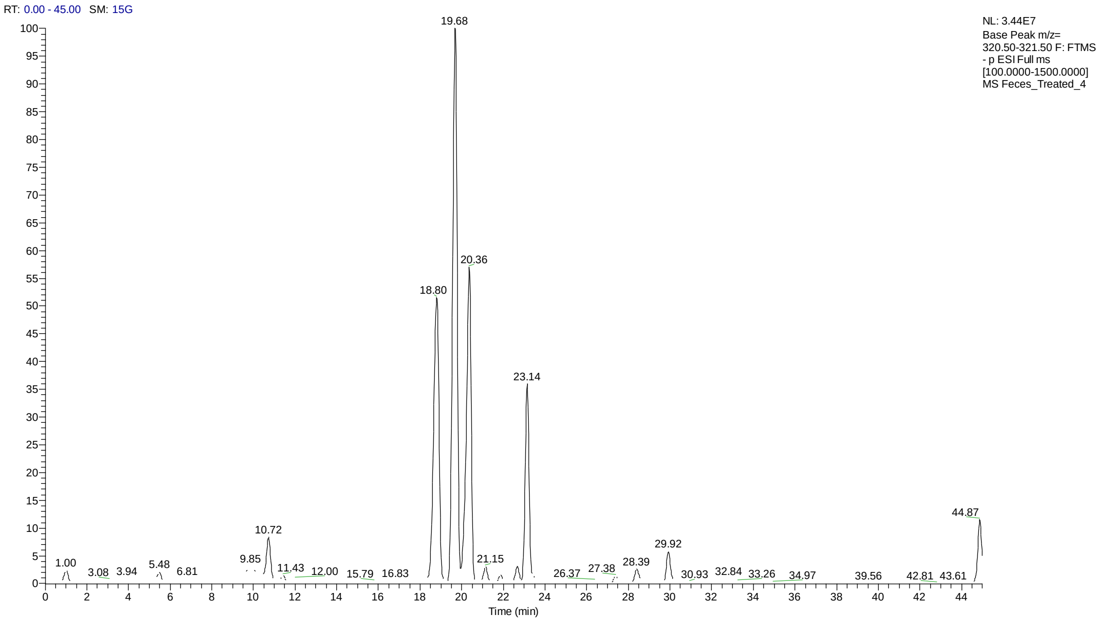

Figure S13. LC-MS chromatogram of the metabolite M5.

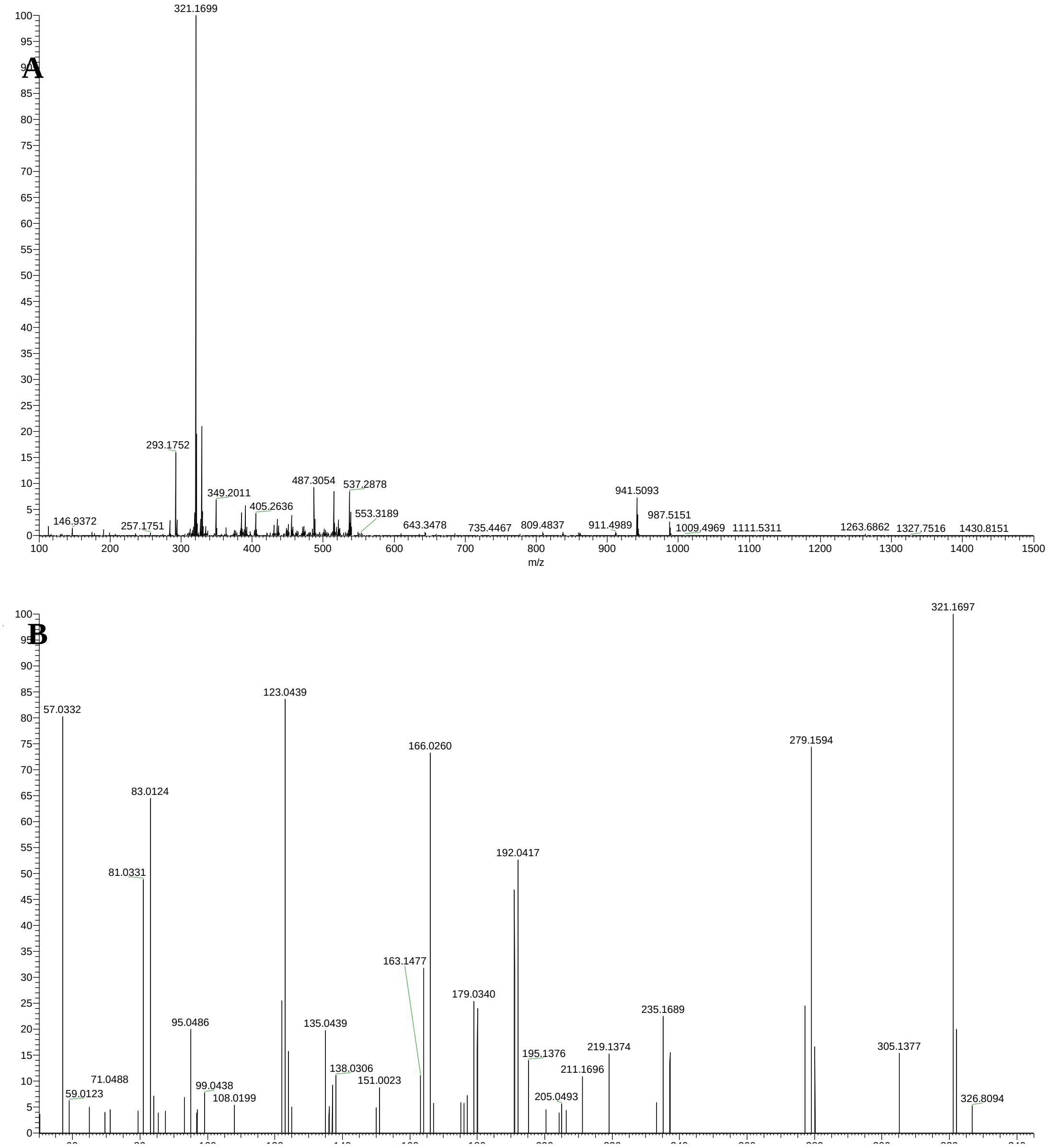

Figure S14. Characterisation of M5 in negative ion mode, (**A**) MS spectrum and (**B**) MS/MS fragmentation spectrum.

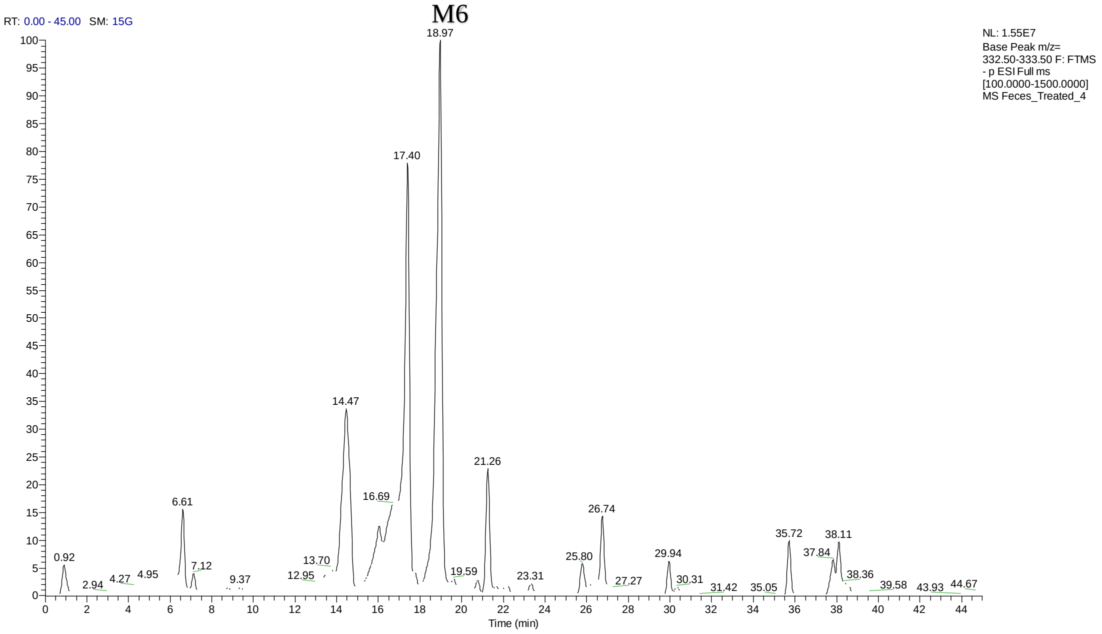

Figure S15. LC-MS chromatogram of the metabolite M6.

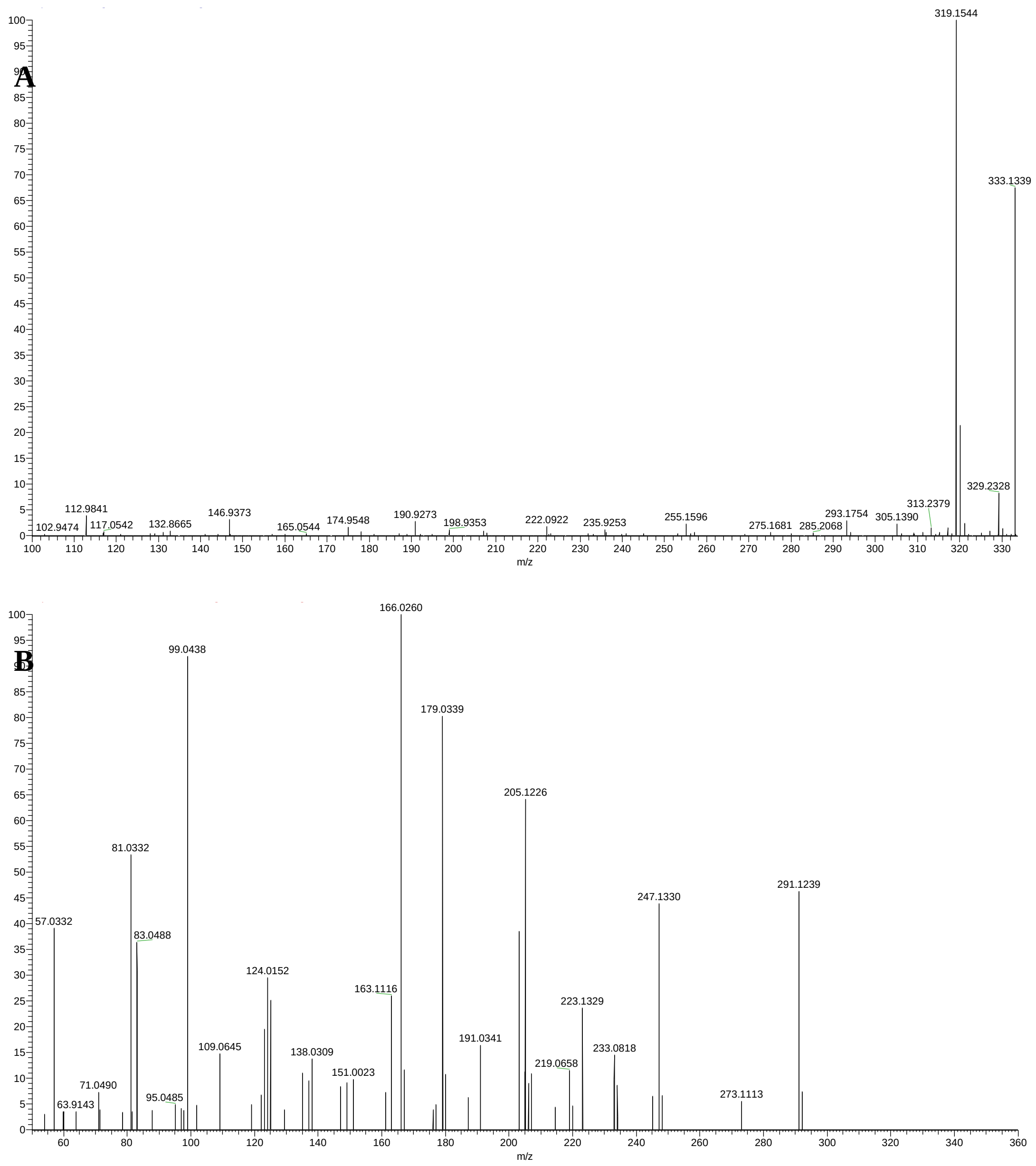

Figure S16. Characterisation of M6 in negative ion mode, (**A**) MS spectrum and (**B**) MS/MS fragmentation spectrum.

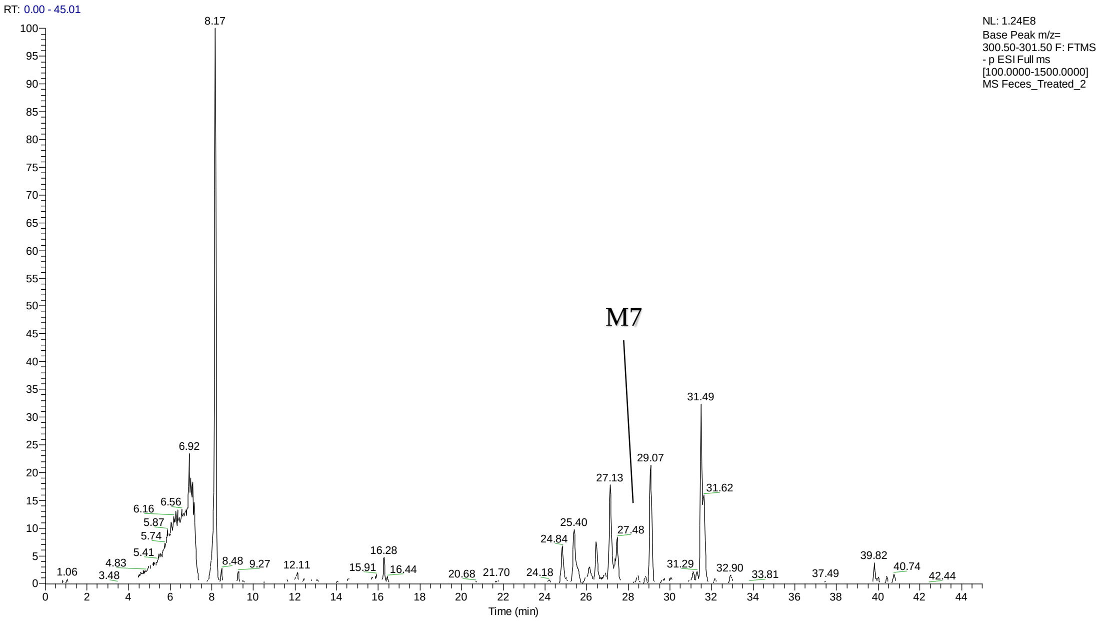

Figure S17. LC-MS chromatogram of the metabolite M7.

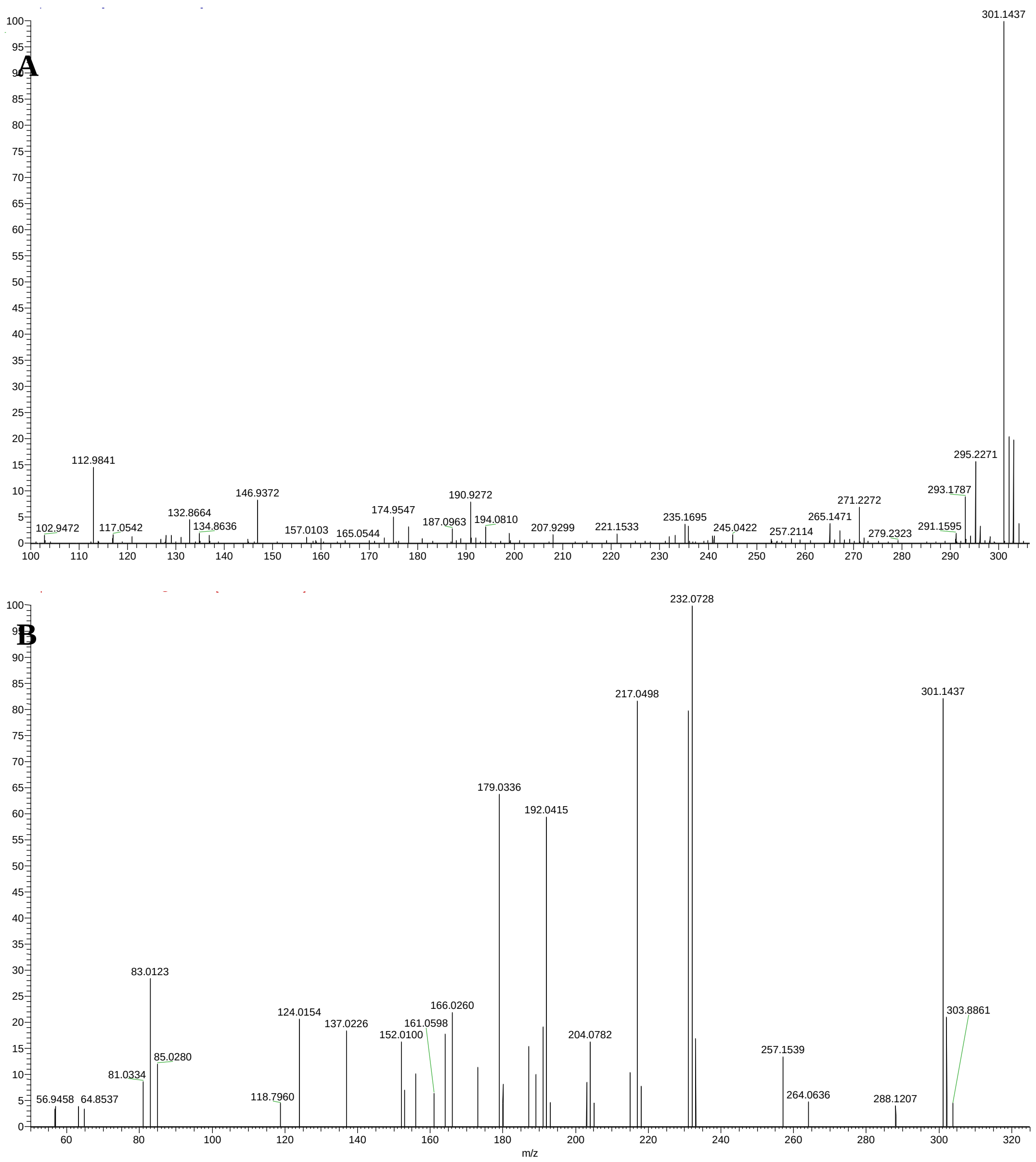

Figure S18. Characterisation of M7 in negative ion mode, (**A**) MS spectrum and (**B**) MS/MS fragmentation spectrum.

M8

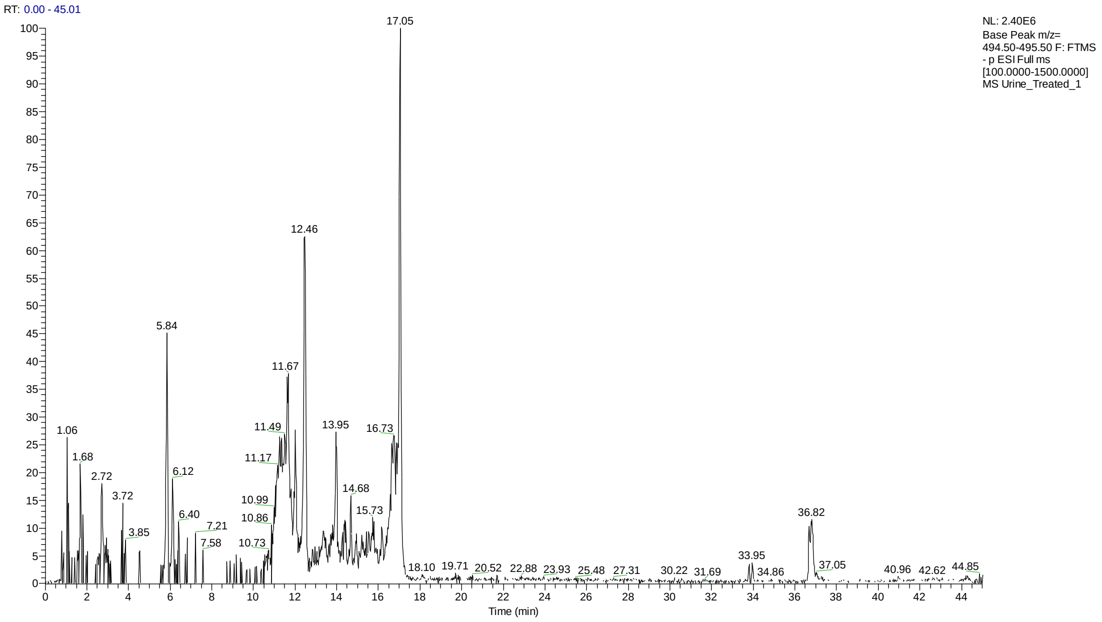

Figure S19. LC-MS chromatogram of the metabolite M8.

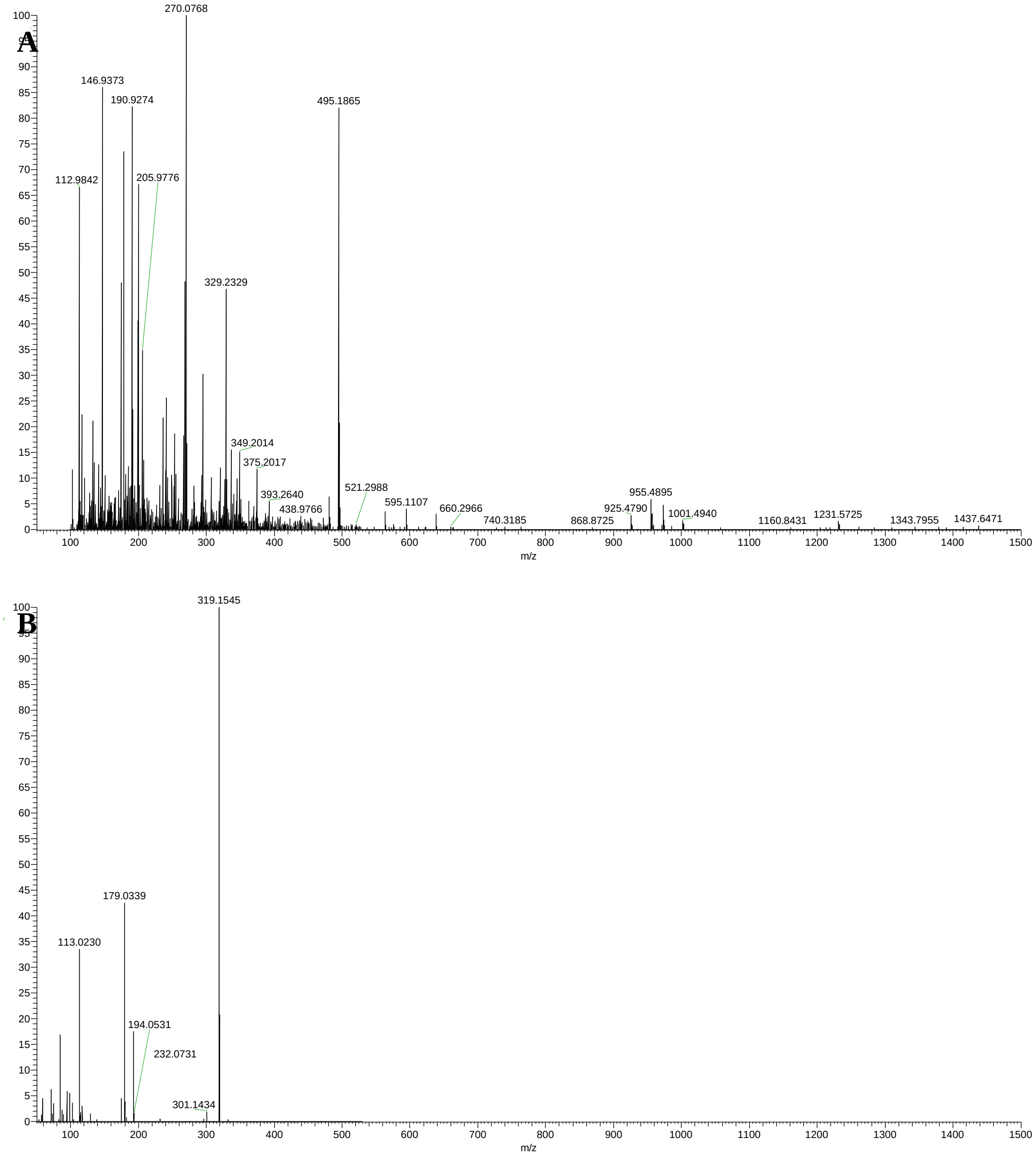

Figure S20. Characterisation of M8 in negative ion mode, (**A**) MS spectrum and (**B**) MS/MS fragmentation spectrum.

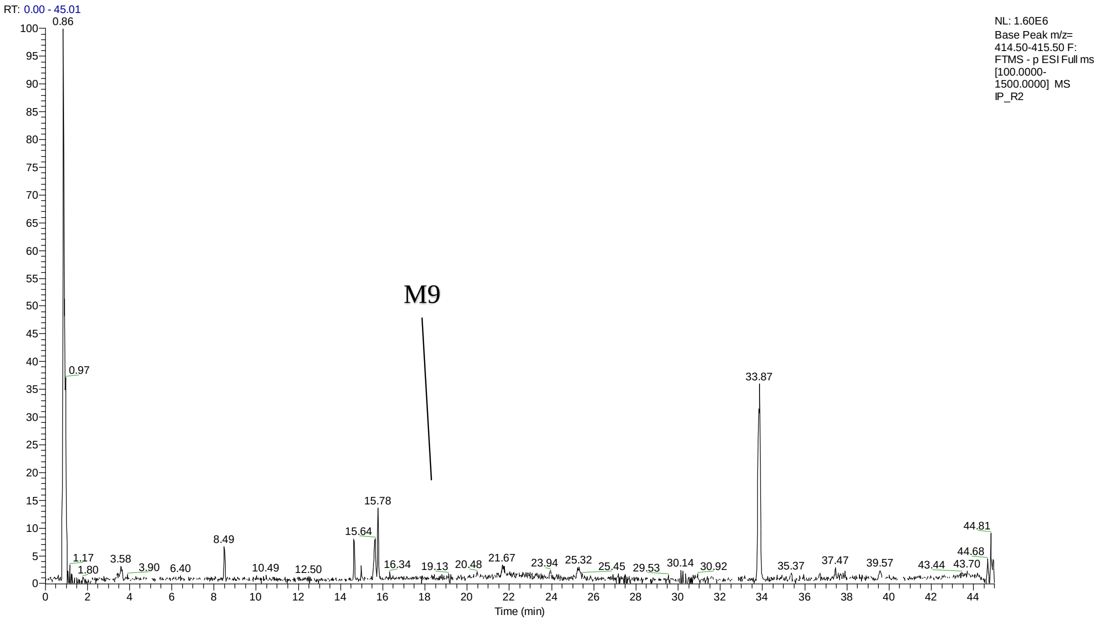

Figure S21. LC-MS chromatogram of the metabolite M9.

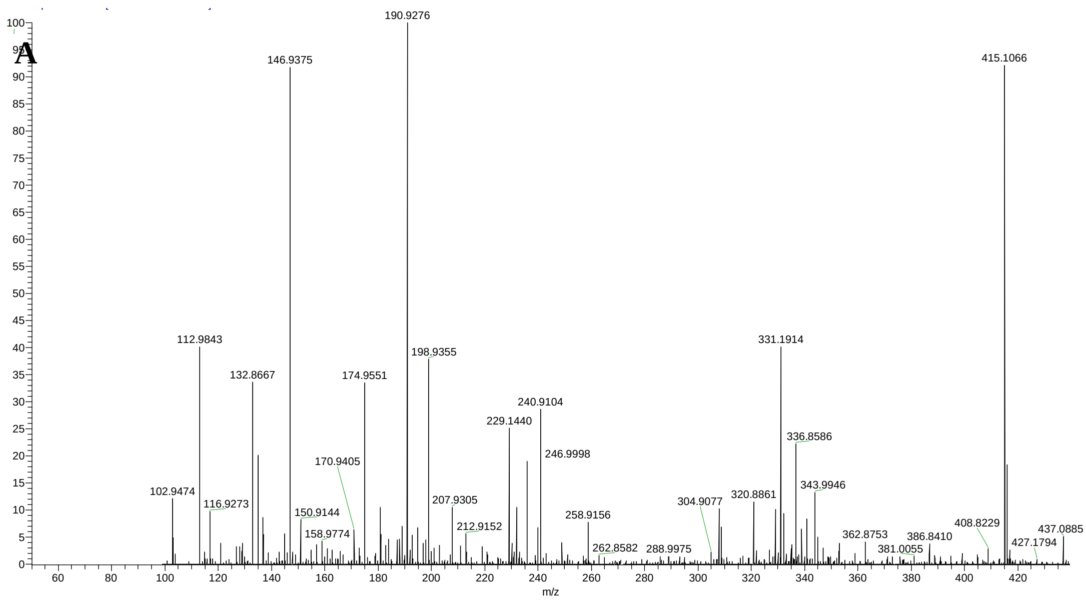

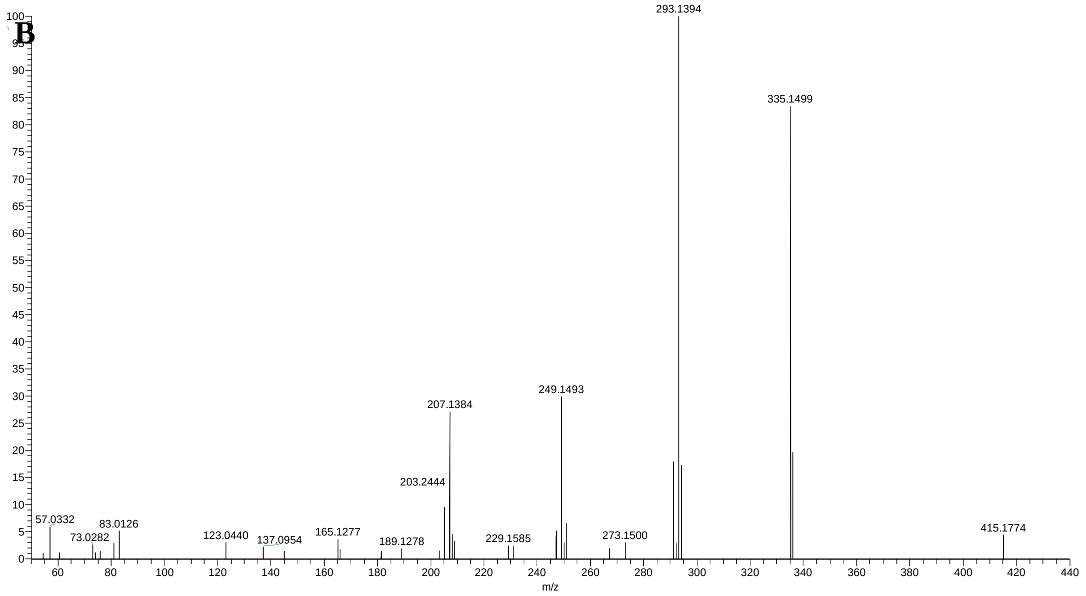

Figure S22. Characterisation of M9 in negative ion mode, (**A**) MS spectrum and (**B**) MS/MS fragmentation spectrum.

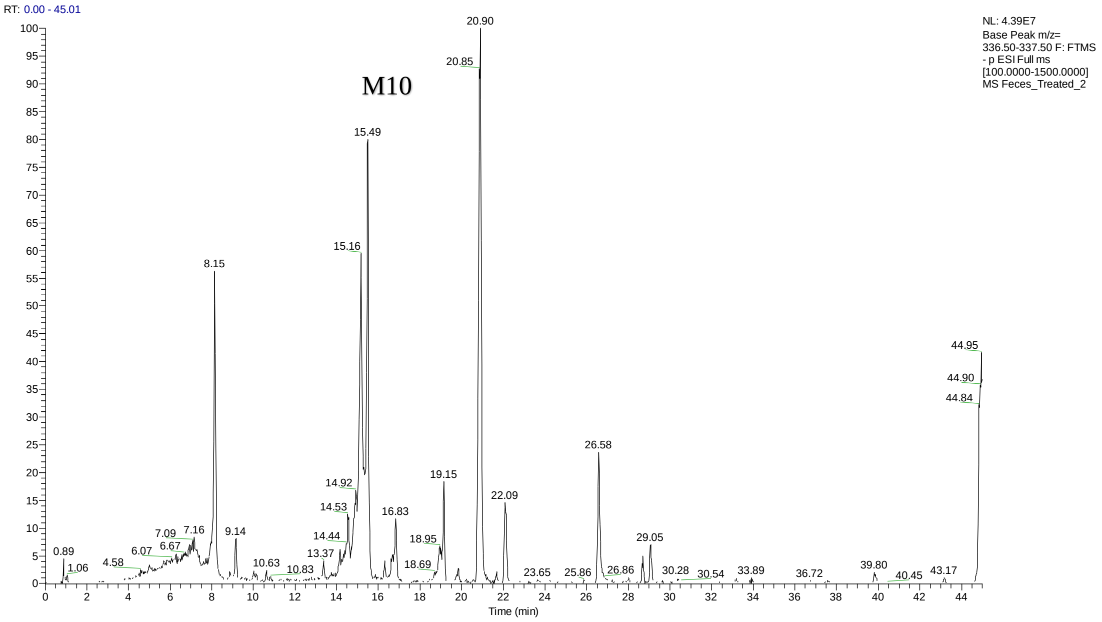

Figure S23. LC-MS chromatogram of the metabolite M10.

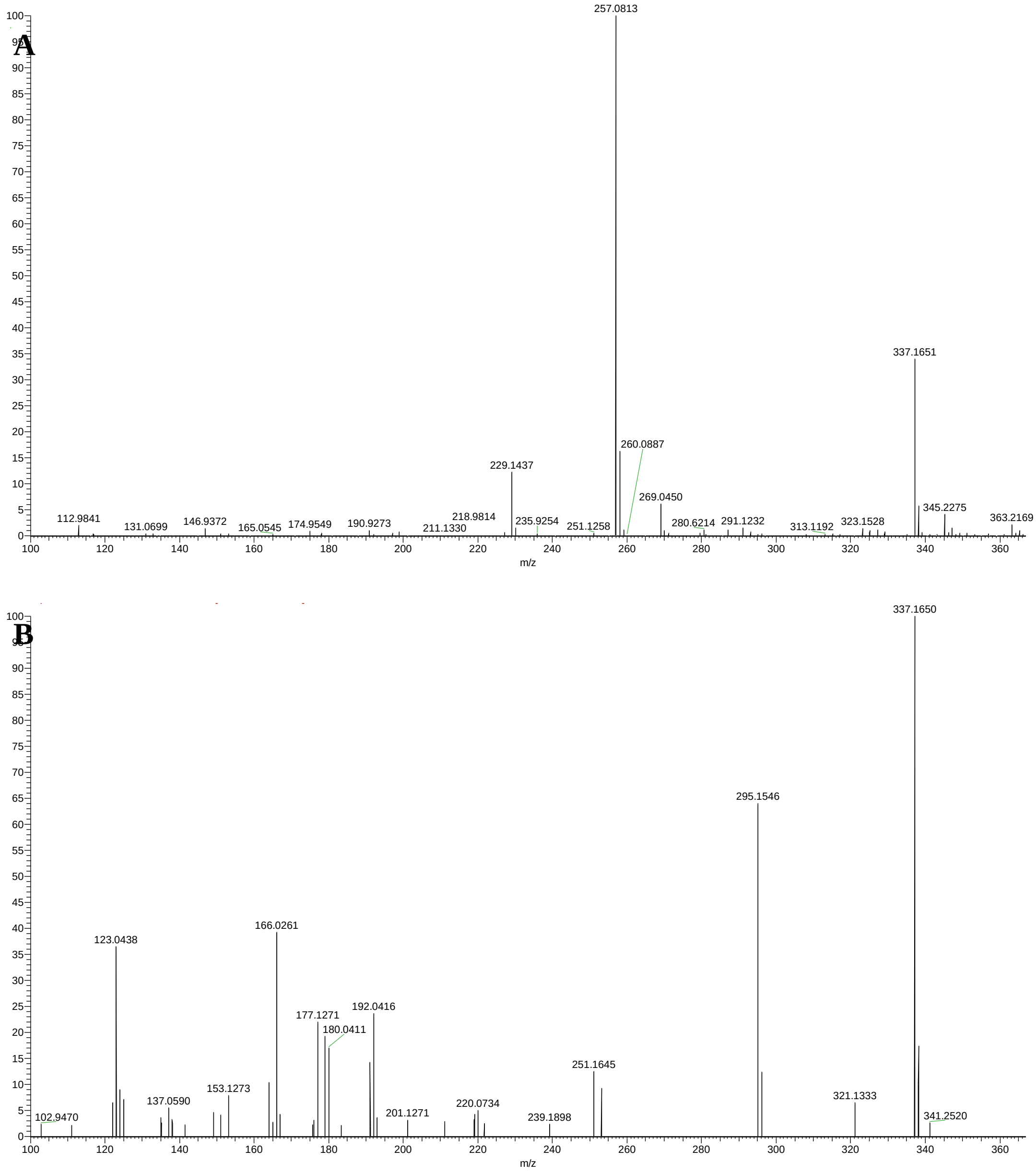

Figure F24. Characterisation of M10 in negative ion mode, (**A**) MS spectrum and (**B**) MS/MS fragmentation spectrum.

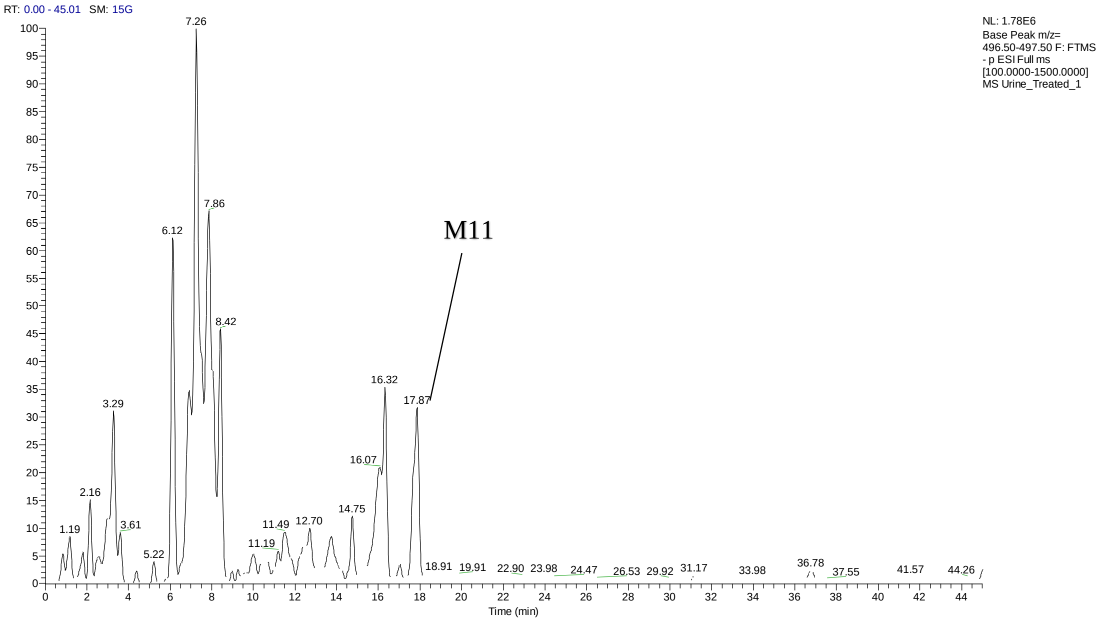

Figure S25. LC-MS chromatogram of the metabolite M11.

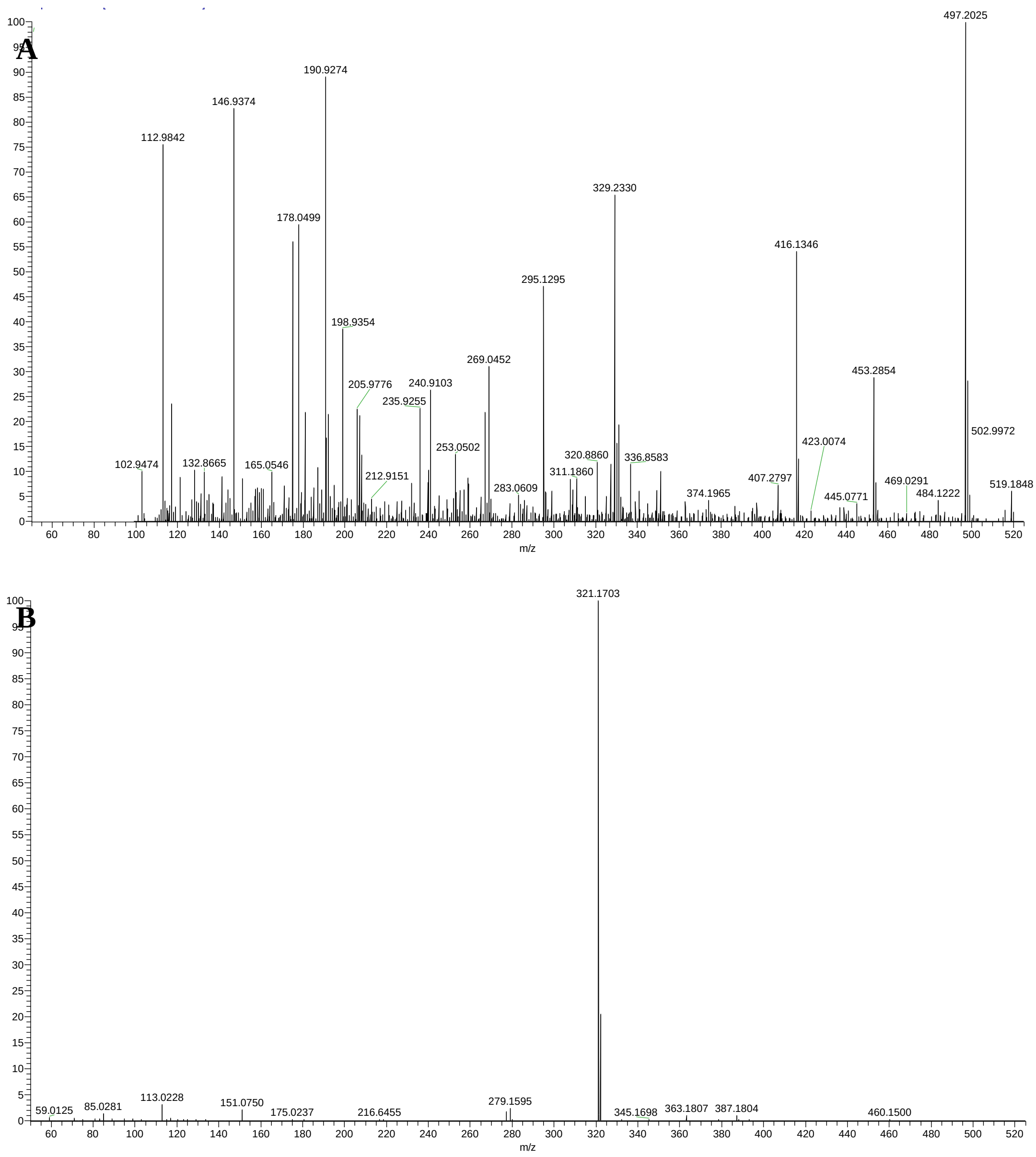

Figure S26. Characterisation of M11 in negative ion mode, (**A**) MS spectrum and (**B**) MS/MS fragmentation spectrum.

M12

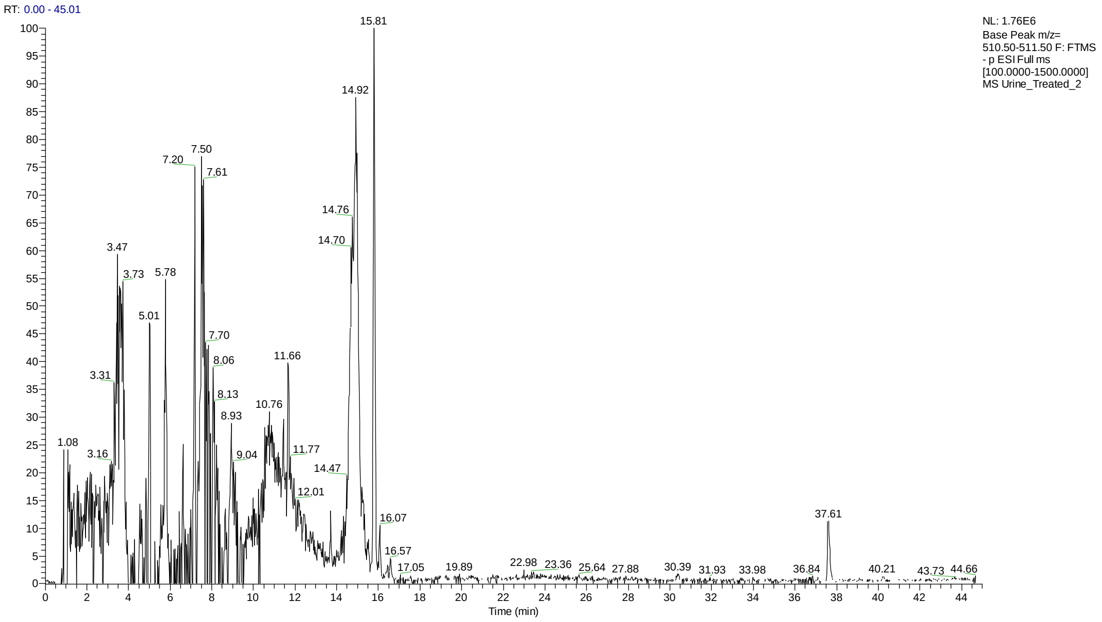

Figure S27. LC-MS chromatogram of the metabolite M12.

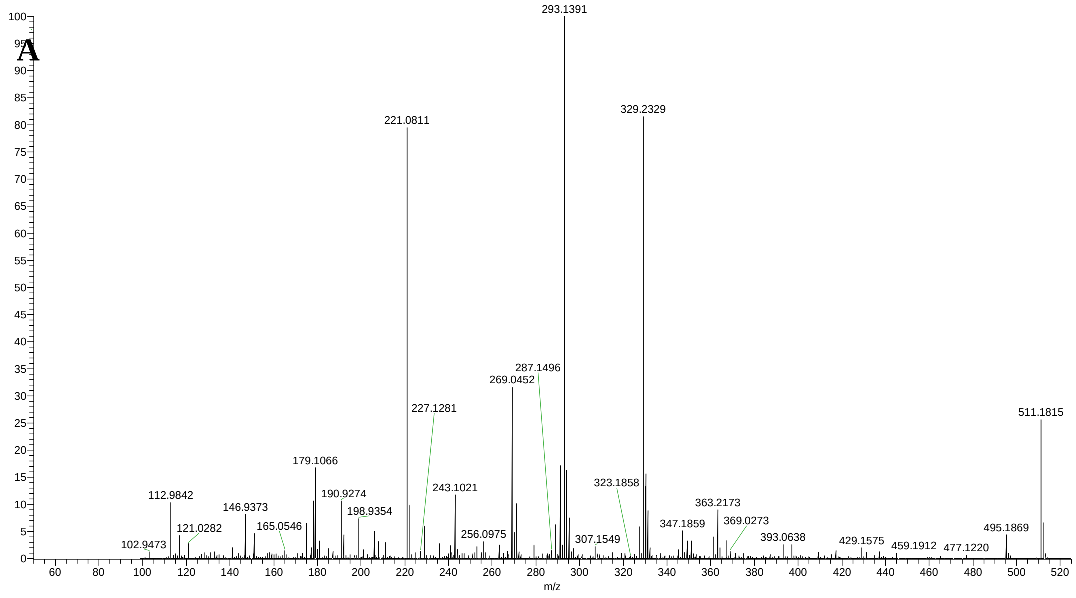

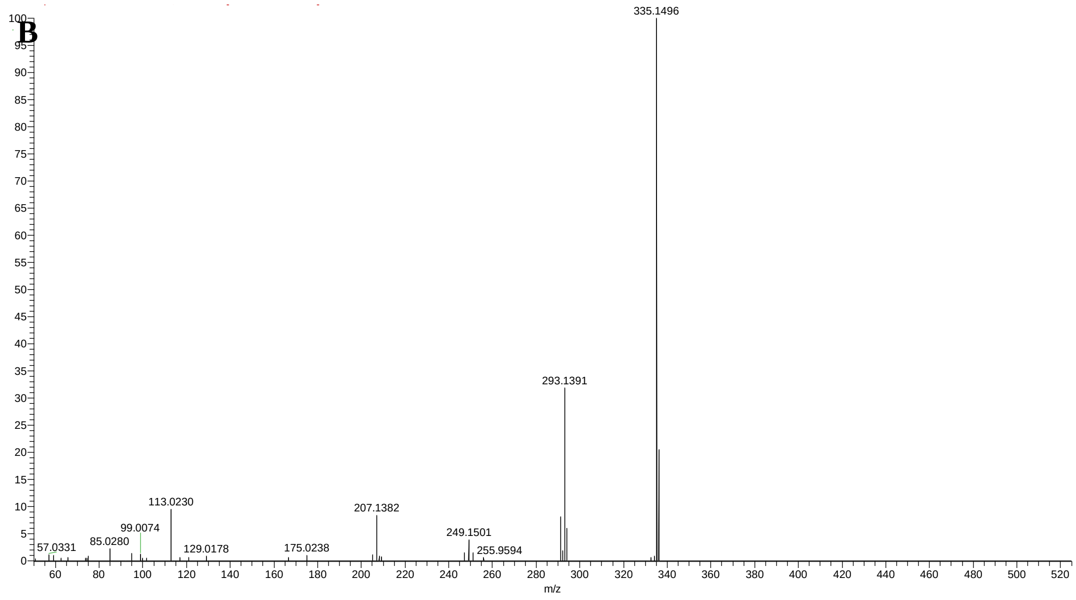

Figure S28. Characterisation of M12 in negative ion mode, (**A**) MS spectrum and (**B**) MS/MS fragmentation spectrum.
